# Reconstructing gene network structure and dynamics from single cell data

**DOI:** 10.1093/bioinformatics/btaf598

**Published:** 2025-11-03

**Authors:** Feng Chen, Chunhe Li

**Affiliations:** Institute of Science and Technology for Brain-Inspired Intelligence, Fudan University, Shanghai 200433, China; Shanghai Center for Mathematical Sciences, Fudan University, Shanghai 200438, China; Institute of Science and Technology for Brain-Inspired Intelligence, Fudan University, Shanghai 200433, China; Shanghai Center for Mathematical Sciences, Fudan University, Shanghai 200438, China; School of Mathematical Sciences, Shanghai Key Laboratory for Contemporary Applied Mathematics and MOE Frontiers Center for Brain Science, Fudan University, Shanghai 200433, China

## Abstract

**Motivation:**

Biological functions are governed by gene regulatory networks (GRNs). Accurately inferring GRNs from high-dimensional and noisy single-cell data remains a major challenge in systems biology. Conventional approaches often struggle with robustness and interpretability, particularly when applied to complex biological processes such as cell fate decisions and complex diseases.

**Results:**

In this study, we propose GGANO, a hybrid framework that integrates Gaussian Graphical Models for conditional independence learning with Neural Ordinary Differential Equations for dynamic modeling and inference. Benchmark analyzes show that GGANO achieves superior accuracy and stability compared to existing methods, particularly under high-noise conditions. Furthermore, GGANO enables the inference of stochastic dynamics from single-cell data. Applying GGANO to the EMT datasets, we uncover intermediate cellular states and key regulatory genes driving EMT progression.

**Availability and implementation:**

The source code is available at GitHub: https://github.com/ChenFeng87/GGANO.

## Introduction

Biological functions are carried out by gene regulatory networks. The mapping between the interaction network topology and its function is one of the central themes in systems biology. Therefore, reconstructing gene regulatory networks (GRNs) and their corresponding dynamics from biological data is crucial to unraveling the complex mechanisms that govern these processes (Su *et al.* 2022, Bocci *et al.* 2022).

For smaller networks of lower complexity, some researchers have obtained satisfactory results using exhaustive search approaches (Chau *et al.* 2012, Hornung and Barkai 2008, Li *et al.* 2017, Ma *et al.* 2006, 2009). However, for larger and more complex systems, the search space grows exponentially as the network size increases. To address this issue, some researchers have tried to build large networks using known small modules (Qiao *et al.* 2019, Xiong *et al.* 2016). However, these methods require certain prior knowledge of the small modules, such as their connection patterns and the functions they exert in the network. In our previous research, we used the ensemble concept to improve the stability of the model (Chen and Li 2022). Nevertheless, this approach is primarily limited to low-dimensional networks, as model training typically requires substantial data. When only sparse and incomplete descriptions of the target function are available, identifying the most probable trajectories (i.e. interpolating the data) is itself a challenging task. Consequently, developing network inference methods tailored for high-dimensional systems has become an urgent and critical objective.

Core GRNs can also be constructed through extensive literature mining. Although this approach works well in systems with substantial accumulation of knowledge, its applicability is limited for systems where key genes and regulatory interactions have not yet been identified. The rapid growth of biomedical publications has made manual curation of literature information extremely time-consuming and prone to human errors. To address these issues, researchers often rely on existing curated databases such as KEGG (Kanehisa *et al.* 2021) and Ingenuity Pathway Analysis (IPA) (Krämer *et al.* 2014). However, these databases encounter challenges when dealing with context-specific interactions. For example, in the process of epithelial-mesenchymal transition (EMT), studies have shown that marker genes for epithelial and mesenchymal states exhibit significant environmental specificity (Cook and Vanderhyden 2020, Peixoto *et al.* 2019, McFaline-Figueroa *et al.* 2019). As a result, relying solely on a limited subset of these marker genes to determine whether a cell is in an epithelial or mesenchymal state may lead to erroneous conclusions. Moreover, with the rapid advancement of biotechnology, the collection of single-cell data has become increasingly abundant (Tang *et al.* 2009). Yet, due to technical and biological limitations, noise and outliers are inevitably present in the data (Akers and Murali 2021). Therefore, the challenges of specificity and noise in the data remain critical obstacles to accurately building GRNs.

In this study, we introduce GGANO that combines the Gaussian graph model (GGM) (Lauritzen 1996) and the Neural ordinary differential equation (Neural ODE) model (Chen *et al.* 2018) to infer gene regulatory networks from high-dimensional data. GGM infers the undirected structure of a regulatory network by leveraging conditional independence relationships between variables. Specifically, it can be proven that two variables $X_{i}$ and $X_{j}$ are conditionally independent if and only if the corresponding element in the precision matrix, $\Theta_{ij}$, is zero. This indicates that the inference of the undirected graph is equivalent to the identification problem of zero elements in the concentration matrix (Edwards 2000, Lauritzen 1996, Whittaker 2009). We incorporate Lasso regularization into the GGM to enhance the sparsity of the network structure (Friedman *et al.* 2008, Yuan and Lin 2007). Considering the stability of biological networks, we introduce a penalty inspired by Fused Lasso (Monti *et al.* 2014, Tibshirani *et al.* 2005), which effectively constrains the differences between consecutive networks, thereby ensuring temporal homogeneity. Previous studies have demonstrated the success of the Neural ODE in network inference and modeling biological processes (Chen and Li 2022, Roesch *et al.* 2021). Here, we use the undirected graph structure derived from the GGM as a prior constraint for the Neural ODE model, enabling further inference of the direction and type of regulatory interactions. This approach not only reduces the search space for neural network parameters, but also reduces the requirements for dataset size during training process.

To demonstrate the advantages of GGANO, we first illustrate its basic idea using a synthetic network. We then focus on some specific gene regulatory systems with multistability, including a Mouse Embryonic Stem Cell (MESC) network (Dunn *et al.* 2014) and a 52-dimensional Human Embryonic Stem Cell (HESC) network (Li and Wang 2013). To assess the accuracy of predicted regulatory interactions, we use metrics such as Receiver Operating Characteristic (ROC) curve, Area Under Curve (AUC), F1-score, and precision. We compare GGM with existing network inference algorithms, including PCM (Leng *et al.* 2020), GENIE3 (Huynh-Thu *et al.* 2010), and GRNBoost2 (Moerman *et al.* 2019), and further evaluate GGANO against the pure Neural ODE approach without prior structural constraints. GGANO achieves superior accuracy and robustness in network inference, even under high-noise conditions. We further apply GGANO to two types of single-cell datasets to assess its effectiveness in realistic data. For the datasets of early mouse embryonic development, we infer the structure of regulatory networks and identify interactions that are specifically associated with the two successive cell fate decisions. For EMT datasets, based on single-gene knockout experiments performed on the Neural ODE model, we predict key genes that promote EMT, which are supported by experimental evidence.

Beyond network inference, there is another key problem in systems biology, which is to gain insights into the stochastic dynamics of gene regulatory systems. The energy landscape theory offers a promising framework for addressing this question (Ge and Qian 2016, Jiang *et al.* 2022, Li and Wang 2013, Zhou *et al.* 2021). For example, in our previous work, we developed a self-consistent mean field approximation (PSCA) approach to calculate the steady state probability distribution and potential energy landscape (Kang *et al.* 2019), and a model-based dimension reduction approach of the landscape (DRL) for high-dimensional gene regulatory systems (Kang and Li 2021). However, these methods are limited by the choice of explicit modeling functions and the accuracy of parameter estimation. Recently, a growing number of data-driven methods have emerged (Chen *et al.* 2019, Guo and Zheng 2017, Jiang *et al.* 2022, Zhang *et al.* 2019, Zhou *et al.* 2021), but these approaches usually lack interpretations of molecular regulations. In this work, we propose a hybrid strategy that combines GGANO with the DRL approach to quantify the energy landscape of gene networks. Applying this strategy, we identify an intermediate, partial-EMT cellular state characterized by pronounced plasticity. For tumor cells in the partial EMT state, they possess relatively high plasticity. On the one hand, it is manifested that they have a higher metastasis risk compared to the E state and the M state. This is because cancer cells in the partial EMT state can undergo collective migration through their epithelial traits and enhance the attachment to the extracellular matrix through their mesenchymal properties, thereby facilitating the detachment of cancer cells from the primary site and the invasion of adjacent tissues (Saitoh 2018). These findings advance our mechanistic understanding of the EMT process and provide theoretical support for clinical cancer research and therapeutic development.

## Materials and methods

### Data overview

In this study, we analyze two single-cell datasets. The first dataset consists of scRNA-seq data generated using MULTI-seq technology (McGinnis *et al.* 2019) from 12 EMT time-course experiments (Cook and Vanderhyden 2020), involving four cancer cell lines: A549 (lung), DU145 (prostate), MCF7 (breast), and OVCA420 (ovarian). Each line was treated with three established EMT-inducing factors (TGF β1, EGF, and TNF). These cell lines, which exhibit epithelial morphology in vitro, have been shown to undergo EMT in previous studies (Dalmau *et al.* 2015, Dong *et al.* 2007, Kasai *et al.* 2005, Li *et al.* 2015, Lu *et al.* 2003, Osborne *et al.* 2014, Sun *et al.* 2018), and represent four distinct cancer types. Detailed information on the data set, including cell numbers per condition, gene filtering criteria, and normalization steps, is provided in Supplementary Material: Section 1. The second dataset comprises single-cell RNA-seq data from pre-implantation mouse embryonic development, as described in Supplementary Material: Section 8.

### The temporal Gaussian graphical model with multiple conditions

In general, it is supposed that *r* stimulation experiments have been carried out for the same cell line. For the *i*-th time point of the *j*-th induction experiment, there are $n_{ij}$ sampling points, namely $\left\{X_{1}^{\left(i,j\right)},X_{2}^{\left(i,j\right)},\ldots ,X_{n_{ij}}^{\left(i,j\right)}\right\}$, where j∈{1,2,…,r}, i∈{1,2,…,T}. We assume that $X_{k}^{\left(i,j\right)}$ follows the multivariate Gaussian distribution, $X_{k}^{\left(i,j\right)}∽N(\mu^{\left(i,j\right)},\Sigma^{\left(i,j\right)})$. Our goal is to infer the undirected regulatory networks that vary over time under each induction condition by estimating the corresponding precision matrices $\left\{\Theta_{ij}\}=\{\Theta_{11},\Theta_{21},\ldots ,\Theta_{T1},\Theta_{12},\Theta_{22},\ldots ,\Theta_{Tr}\right\}$. Here, $\Theta_{ij}$ encodes the partial correlation structure at the *i*-th time point under the *j*-th inducer condition. It follows that we can encode $\Theta_{ij}$ as a graph or network $G_{ij}$, where the existence of an edge indicates a non-zero entry in the corresponding precision matrix and can be construed as a functional relationship between the two nodes in question. To ensure the sparsity and temporal homogeneity of the graph $G_{ij}$, we formulate the following optimization problem (Supplementary Material: Section 3):


(1) $$\begin{array}{cc} \underset{\Theta_{1},\Theta_{ij}}{\min} & -n_{1}\left(\text{log}\;\det\Theta_{1}-\mathrm{tr}(S_{1}\Theta_{1})\right)+\lambda ||\Theta_{1}|{|}_{\text{od},1}\\ & +\sum_{j=1}^{r}\sum_{i=2}^{T}[-n_{ij}\left(\text{log}\;\det\Theta_{ij}-\mathrm{tr}(S_{ij}\Theta_{ij})\right)+\lambda ||\Theta_{ij}|{|}_{\text{od},1}]\\ & +\sum_{j=1}^{r}\sum_{i=2}^{T}\beta \frac{||\Theta_{ij}-\Theta_{i-1,j}|{|}_{F}^{2}}{h_{ij}},\\ s.t.\; & \Theta_{1}\in S_{≻0}^{N},\\ & \Theta_{ij}\in S_{≻0}^{N},\;\forall i\in \{2,\ldots ,T\},\forall j\in \{1,\ldots ,r\}. \end{array}$$

where $S_{≻0}^{N}$ denotes the set of all symmetric positive definite matrices of size N×N. $\Theta_{1}$ represents the precision matrix at the initial time point, with $\Theta_{1}=\Theta_{11}=\Theta_{12}=\ldots =\Theta_{1r}$, and $\Theta_{ij}$ represents the precision matrix at time point *i* under condition *j*. $S_{1}$ and $S_{ij}$ are the corresponding sample covariance matrices. ${\left|\left|\cdot \right|\right|}_{F}$ denotes the Frobenius norm, and ${\left|\left|\cdot \right|\right|}_{\text{od},1}$ is the seminorm, calculated as the sum of the absolute values of all off-diagonal elements. λ≥0 is a parameter governing sparsity, while β regulates the temporal homogeneity. $h_{ij}$ represents the interval between the *i*-th time point and its predecessor under condition *j*.

Consequently, the first term optimizes the initial precision matrix $\Theta_{1}$, the second term estimates $\Theta_{ij}$, and the third constrains similarity between adjacent time points. Lasso regularization further prevents overfitting by limiting the complexity of the model.

Then, we employ the Alternating Direction Method of Multipliers (ADMM) algorithm (Boyd *et al.* 2010) to address this optimization problem. Firstly, to leverage the separability of the objective function, we introduce auxiliary variables *Y* and *Z*, where $Y=\{Y_{1},Y_{21},Y_{22},\ldots ,Y_{2r},Y_{31},Y_{32},\ldots ,Y_{Tr}\}$ and $Z=\{Z_{21},Z_{22},\ldots ,Z_{2r},Z_{31},Z_{32},\ldots Z_{Tr}\}$. Then, we can write the equivalent problem of the above optimization problem:


(2) $$\begin{array}{c} \begin{array}{cc} \underset{\Theta_{1},\Theta_{ij},Y_{1},Y_{ij},Z_{ij}}{\min} & -n_{1}(\text{log}\;\det\Theta_{1}-\mathrm{tr}(S_{1}\Theta_{1}))+\lambda ||Y_{1}|{|}_{\text{od},1}\\ & +\sum_{j=1}^{r}\sum_{i=2}^{T}(-n_{ij}(\;\text{log}\;\det\Theta_{ij}-\mathrm{tr}(S_{ij}\Theta_{ij}))\\ & \;+\lambda ||Y_{ij}|{|}_{\text{od},1}+\beta \frac{||Z_{ij}|{|}_{F}^{2}}{h_{ij}}), \end{array}\\ \begin{array}{cc} s.t. & \Theta_{1}\in S_{≻0}^{N},\\ & Y_{1}=\Theta_{1},\\ & \Theta_{ij}\in S_{≻0}^{N},\;\forall i=2,\ldots ,T,\;\forall j=1,\ldots ,r,\\ & (Y_{i1},\ldots ,Y_{ir})=(\Theta_{i1},\ldots ,\Theta_{ir}),\;\forall i=2,\ldots ,T,\\ & (Z_{i1},\ldots ,Z_{ir})=(\Theta_{i1}-\Theta_{i-1,1},\ldots ,\Theta_{ir}-\Theta_{i-1,r}),\\ & \;\forall i=2,\ldots ,T. \end{array} \end{array}$$

The application of the ADMM algorithm demands the formulation of the augmented Lagrangian related to Eq. 2. It is defined as:


(3) $$\begin{array}{cc} & L_{\rho }(\Theta ,Y,Z,U,V)\\ & =-n_{1}(\text{log}\;\det\Theta_{1}-\mathrm{tr}(S_{1}\Theta_{1}))+\lambda ||Y_{1}|{|}_{\text{od},1}\\ & \;+\sum_{j=1}^{r}\sum_{i=2}^{T}(-n_{ij}(\;\text{log}\;\det\Theta_{ij}-\mathrm{tr}(S_{ij}\Theta_{ij}))\\ & \;+\lambda ||Y_{ij}|{|}_{\text{od},1}+\beta \frac{||Z_{ij}|{|}_{F}^{2}}{h_{ij}})\\ & \;+\frac{\rho }{2}(||\Theta_{1}-Y_{1}+{\tilde{U}}_{1}|{|}_{F}^{2}-||{\tilde{U}}_{1}|{|}_{F}^{2})\\ & \;+\frac{\rho }{2}\sum_{j=1}^{r}\sum_{i=2}^{T}\left(||\Theta_{ij}-Y_{ij}+{\tilde{U}}_{ij}|{|}_{F}^{2}-||{\tilde{U}}_{ij}|{|}_{F}^{2}\right)\\ & \;+\frac{\rho }{2}\sum_{j=1}^{r}\sum_{i=2}^{T}\left(||\Theta_{ij}-\Theta_{i-1,j}-Z_{ij}+{\tilde{V}}_{ij}|{|}_{F}^{2}-||{\tilde{V}}_{ij}|{|}_{F}^{2}\right). \end{array}$$

where ${\tilde{U}}_{ij}=\frac{U_{ij}}{\rho }$, ${\tilde{V}}_{ij}=\frac{V_{ij}}{\rho }$, $U_{ij}$ and $V_{ij}$ are respectively the Lagrange multipliers of $Y_{ij}$ and $Z_{ij}$, and ρ is a positive penalty parameter. The proposed estimation procedure minimizes $L_{\rho }(\Theta ,Y,Z,\tilde{U},\tilde{V})$ through iterative updates of the variables $\Theta ,Y,Z,\tilde{U},\tilde{V}$, and the update formula is:


(4) $$\begin{array}{cc} \Theta_{ij}^{k+1}=\frac{1}{4\eta }Q(-\Lambda +\sqrt{\Lambda^{2}+8\eta I})Q^{T}, & \\ \forall i\in \{1,2,\ldots ,T\},\forall j\in \{1,2,\ldots ,r\}, & \\ Y_{1}^{k+1}=\mathrm{sign}(\Theta_{1}^{k+1}+U_{1}^{k})*\max(|\Theta_{1}^{k+1}+U_{1}^{k}|-\frac{\lambda }{\rho },0), & \\ Y_{ij}^{k+1}=\mathrm{sign}(\Theta_{ij}^{k+1}+U_{ij}^{k})*\max(|\Theta_{ij}^{k+1}+U_{ij}^{k}|-\frac{\lambda }{\rho },0), & \\ \forall i\in \{2,3,\ldots ,T\},\forall j\in \{1,2,\ldots ,r\}, & \\ Z_{ij}^{k+1}=\frac{\rho h_{ij}}{\rho h_{ij}+2\beta }(\Theta_{ij}^{k+1}-\Theta_{i-1,j}^{k+1}+{\tilde{V}}_{ij}^{k}), & \\ \forall i\in \{2,3,\ldots ,T\},\forall j\in \{1,2,\ldots ,r\}, & \\ {\tilde{U}}_{1}^{k+1}={\tilde{U}}_{1}^{k}+\rho (\Theta_{1}^{k+1}-Y_{1}^{k+1}), & \\ {\tilde{U}}_{ij}^{k+1}={\tilde{U}}_{ij}^{k}+\rho (\Theta_{ij}^{k+1}-Y_{ij}^{k+1}), & \\ \forall i\in \{2,3,\ldots ,T\},\forall j\in \{1,2,\ldots ,r\}, & \\ {\tilde{V}}_{ij}^{k+1}={\tilde{V}}_{ij}^{k}+\rho (\Theta_{ij}^{k+1}-\Theta_{i-1,j}^{k+1}-Z_{ij}^{k+1}), & \\ \forall i\in \{2,3,\ldots ,T\},\forall j\in \{1,2,\ldots ,r\}, & \end{array}$$

where $Q\Lambda Q^{T}$ is the eigendecomposition of $S_{ij}-\eta (A+A^{T})$, and


$$\{\begin{array}{c} \eta =\frac{2\rho }{n_{1}},\;A=\frac{\left(Y_{1}^{k}-{\tilde{U}}_{1}^{k})+(\Theta_{21}^{k}-Z_{21}^{k}+{\tilde{V}}_{21}^{k})+(\Theta_{22}^{k}-Z_{22}^{k}+{\tilde{V}}_{22}^{k})+(\Theta_{23}^{k}-Z_{23}^{k}+{\tilde{V}}_{23}^{k}\right)}{4}\\ \text{for}\;i=1,\\ \eta =\frac{3\rho }{2n_{ij}},\;A=\frac{\left(Y_{ij}^{k}-{\tilde{U}}_{ij}^{k})+(\Theta_{i-1,j}^{k+1}+Z_{ij}^{k}-{\tilde{V}}_{ij}^{k})+(\Theta_{i+1,j}^{k}-Z_{i+1,j}^{k}+{\tilde{V}}_{i+1,j}^{k}\right)}{3}\\ \text{for}\;1<i<T,\\ \eta =\frac{\rho }{n_{Tj}},\;A=\frac{\left(Y_{Tj}^{k}-{\tilde{U}}_{Tj}^{k})+(\Theta_{T-1,j}^{k+1}+Z_{Tj}^{k}-{\tilde{V}}_{Tj}^{k}\right)}{2}\\ \text{for}\;i=T. \end{array}$$

### 2.3 Neural ODE modeling

Consider an *N*-dimensional gene regulatory network modeled by differential equations, the *i*-th equation is:


(5) $$\begin{array}{c} \frac{dx_{i}(t)}{dt}=g_{i}(x_{1}(t),x_{2}(t),\ldots ,x_{N}(t))-k_{i}x_{i}(t). \end{array}$$

Here, $x_{i}(t)(i=1,2,\ldots ,N)$ represents the state value of the *i*-th variable at time *t*, $g_{i}$ is a function accounting for the synthesis of $x_{i}$, and $k_{i}$ is the degradation rate. Distinct from traditional neural network models, such as RNNs and CNNs, that focus on the direct modeling of x(t), the Neural ODE in our framework intends to model the generation rate $g=(g_{1},g_{2},\ldots ,g_{N})$, which constitutes a part of the rate of state change. The computational framework we adopt here consists of one input layer, two hidden layers (respectively containing 32 nodes and 128 nodes) and one output layer. The ReLU function is employed as the activation function for the two hidden layers, while the sigmoid function is utilized for the output layer, restricting the state value within the range of 0–1.

For any time *t*, the input of the neural network can encompass other information of the system in addition to the state of each variable x(t) and the prior structure. For example, in the application of EMT data, we add a node to represent the concentration of the inducer. Then, we can obtain the state of the system at time t+Δt via the following discrete iterative equations:


(6) $$\begin{array}{c} x_{i}(t+\Delta t)=x_{i}(t)+g_{i}(t)\Delta t-k_{i}x_{i}(t)\Delta t,\;\;(i=1,2,\ldots ,N). \end{array}$$

In addition, the neural network model is trained through standard gradient optimization (Adam optimizer in Pytorch, with a learning rate of 0.0001), and the minimum batch size is set at 128. The loss function is the norm $L_{1}$, which is the average of the absolute differences between the prediction data and the training data.

## Results

### Overview of GGANO

**Figure 1.**
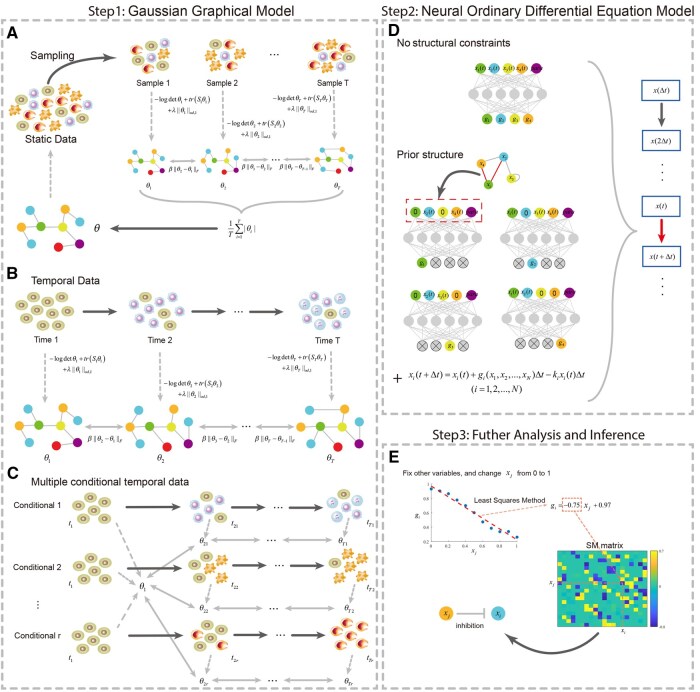
Overview of GGANO for gene network inference. (A) Static single-cell gene expression data analyzed by GGM. Bootstrapping is performed to extract *T* subsets from the data. The network structure is inferred for each subset, and the results are integrated to estimate the underlying regulatory network of the original data. (B) GGM applied to temporal data. (C) GGM applied to multiple conditional temporal datasets. (D) Neural ODE used to model the system. The input includes the state of the variable at time *t* and additional parameter information about the system. The output is the generation rate of the corresponding variable, which is then used in an iterative equation to compute the variable’s state at the next time step. In the absence of prior structural information, it is assumed by default that each variable is connected to all other variables, including itself. When prior structural information is available, the state values of variables that are not connected to the target variable, as indicated by the structure, are set to zero at the input layer, while the state values of other variables remain unchanged. (E) Determining the direction and type of regulatory action from the mathematical properties of the generation rate. To assess the effect of $x_{j}$ on $x_{i}$, all variables except $x_{j}$ are fixed, and the value of $x_{j}$ is gradually increased from 0 to 1. A linear fit of the recorded $g_{i}$ is performed using the least squares method, and the slope of the resulting linear function represents the corresponding element $SM_{ij}$ in the SM matrix. If $SM_{ij}$ is significantly greater than 0, $x_{j}$ has an activating regulatory effect on $x_{i}$. Conversely, if $SM_{ij}$ is significantly less than 0, $x_{j}$ exerts an inhibitory effect on $x_{i}$.

We propose GGANO, a computational framework integrating GGM and Neural ODE to infer regulatory networks and analyze stochastic dynamics. In GGM, conditional independence is used to reconstruct undirected network structures. To improve stability, we adopt a Bagging strategy for static data (Fig. 1A). For temporal data, we assume regulatory networks do not undergo abrupt changes between successive time points (Fig. 1B). For multi-condition data, shared regulatory relationships are extracted by integrating datasets through common initial states, ensuring both consistency and interpretability (Fig. 1C).

Next, we use the Neural ODE to model the system. The inputs include the state values of the variables at each time point, along with additional prior parameters of the system, denoted as para (Fig. 1D). The specific form of para is determined by the experimental context and may represent varying experimental conditions, external input intensities, prior descriptions of system properties (e.g. coupling strength between variables), or other relevant factors. If one wishes to use a unified Neural ODE model to learn different forms of g, para can serve as a label to represent distinct systems, enabling continuous transitions between different system configurations. The outputs correspond to the generation rate g of the variables, and the state values of the variables at the subsequent time point are obtained through an iterative equation.

For the undirected binary graph obtained from GGM, prior structural information can be incorporated into the Neural ODE through a masking strategy. In particular, when computing the generation rate of gene $x_{i}$, the Neural ODE model retains as input only the values of genes that are connected to $x_{i}$ in the undirected graph, while setting the values of all other genes to zero. This ensures that $g_{i}$ reflects only the potential influence of its neighbors in the prior graph. Thus, in principle, for a system with *N* variables, the model needs to be executed at most *N* times to produce all outputs, whereas a standard Neural ODE without structural constraints requires only a single evaluation per step.

Subsequently, by examining the mathematical properties of g (monotonicity), we can further determine the direction and type of the regulatory effects (Fig. 1E). Specifically, for any pair of variables i,j∈{1,2,…,N}, we fix all variables except $X_{j}$, and incrementally increase the value of $X_{j}$ from 0 to 1, while recording the corresponding $g_{i}$ at each step. Here, we normalized the variable range to be *[*0, 1*]*. Alternatively, one may select the interval between the empirical minimum and maximum values of $X_{j}$ observed in the dataset. Then, we perform a linear regression using the least squares method on the recorded data, obtaining the slope of the resulting linear function (denoted as $SM_{ji}$). Therefore, if the slope is negative, it indicates that $X_{j}$ has an inhibitory effect on $X_{i}$; conversely, if the slope is positive, it suggests that $X_{j}$ has an activating effect on $X_{i}$.

### Network inference for the synthetic network

**Figure 2.**
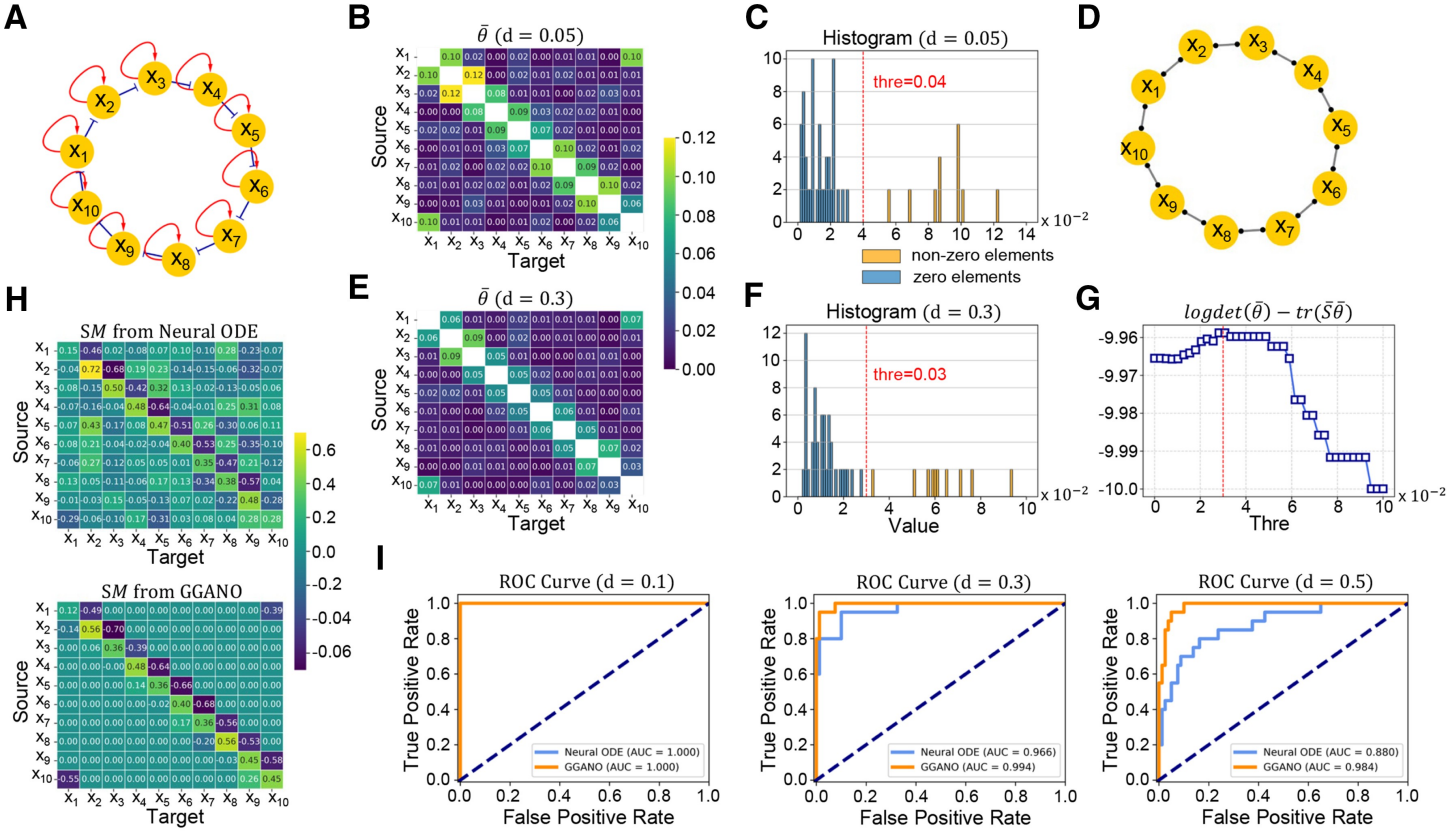
Demonstration of GGANO on synthetic network. (A) Network topology of the ten-node synthetic system. (B) Heatmap of the concentration matrix $\bar{\Theta }$ estimated by GGM with noise level d=0.05. (C) Distribution of the elements of $\bar{\Theta }$ in (B). (D) Undirected graph structure inferred by GGM. (E) Heatmap of $\bar{\Theta }$ estimated by GGM with noise level d=0.3. (F) Distribution of the elements of $\bar{\Theta }$ in (E). (G) Determination of the threshold by maximizing the likelihood function. For $\bar{\Theta }$ in (E), the likelihood reaches its maximum when the threshold is between 0.28 and 0.32. (H) SM matrices obtained using the pure Neural ODE and GGANO, respectively, at noise level d=0.3. (I) ROC curves based on the estimated SM matrices, comparing the inference performance of pure Neural ODE and GGANO under different noise levels.

We demonstrate the effectiveness of GGANO using several simulation datasets. First, using a ten-dimensional synthetic network we illustrate the basic approach (topology in Fig. 2A; mathematical equations in Supplementary Material: Section 2). Following the scheme in Fig. 1A, we randomly extract 20 sampling sets from the generated data, each containing 10,000 points. For each set, the static precision matrix $\Theta_{i}$ is estimated (Supplementary Material: Section 3), and the average $\bar{\Theta }=\frac{1}{20}\sum_{i=1}^{20}|\Theta_{i}|$ is computed. Applying a suitable threshold *thre* to $\bar{\Theta }$ yields the final regulatory network: if ${\bar{\Theta }}_{ij}>\mathrm{thre}$, $X_{i}$ and $X_{j}$ are connected; otherwise, they are considered unconnected.

At low noise (d=0.05), zero and non-zero elements in $\bar{\Theta }$ are clearly separated (Fig. 2B, C), allowing straightforward threshold selection. As noise increases (d=0.3), this separation narrows (Fig. 2E, F). In such cases, the optimal threshold is determined by maximizing the likelihood function (Fig. 2G):


(7) $$\begin{array}{cc} \underset{\mathrm{thre}}{\max} & \;\text{log}\;\det({\bar{\Theta }}_{\mathrm{thre}})-\mathrm{tr}(\bar{S}{\bar{\Theta }}_{\mathrm{thre}}),\\ s.t. & \mathrm{thre}\in [0,1], \end{array}$$

where $\bar{S}=\frac{1}{20}\sum_{i=1}^{20}S_{i}$, and $S_{i}$ is the sample covariance matrix of the *i*-th sampling set. The graph inferred by the GGM is undirected and contains no information regarding the direction and type of regulatory effects. Therefore, We use this undirected network as prior knowledge in the Neural ODE (Fig. 1D) to infer activating or inhibitory relationships based on the monotonicity of the generation rates: if $g_{i}$ increases with $X_{j}$, $X_{j}$ activates $X_{i}$; if $g_{i}$ decreases, $X_{j}$ inhibits $X_{i}$ (Chen and Li 2022).

Compared with the Neural ODE alone, GGANO achieves superior network inference and demonstrates remarkable robustness as data noise increases. For instance, under d=0.3, the pure Neural ODE infers extra regulatory effects (e.g. $X_{2}⊣X_{9}$, $X_{6}⊣X_{9}$, $X_{5}\to X_{2}$) due to multiple parameter configurations generating the same observations (Fig. 2H). This reflects a general feature of biological systems: multiple patterns can realize the same function, and alternative pathways can compensate when one is disrupted. However, the mechanisms underlying such redundancy often remain unclear. Therefore, exploring the key regulatory networks with specific biological functions is exceptionally vital. This not only enhances interpretability but also provides a basis for investigating the cooperative mechanisms between different pathways.

To make comparisons, we plot the Receiver Operating Characteristic (ROC) curves corresponding to the two approaches (Fig. 2I) and calculate the corresponding Area Under Curve (AUC). When the noise level is low, both of the two approaches are capable of precisely inferring the network structure. However, as the noise level rises, the AUC value of GGANO remains relatively stable, whereas the AUC value of the pure Neural ODE undergoes a notable decline (Fig. 2I), suggesting that GGANO outperforms the pure Neural ODE in both accuracy and robustness. And the higher the noise level, the more evident the superiority of GGANO becomes (The results at other noise levels are shown in Supplementary Material: Fig. S1).

### Network inference for the mouse embryonic stem cell (MESC) network

**Figure 3.**
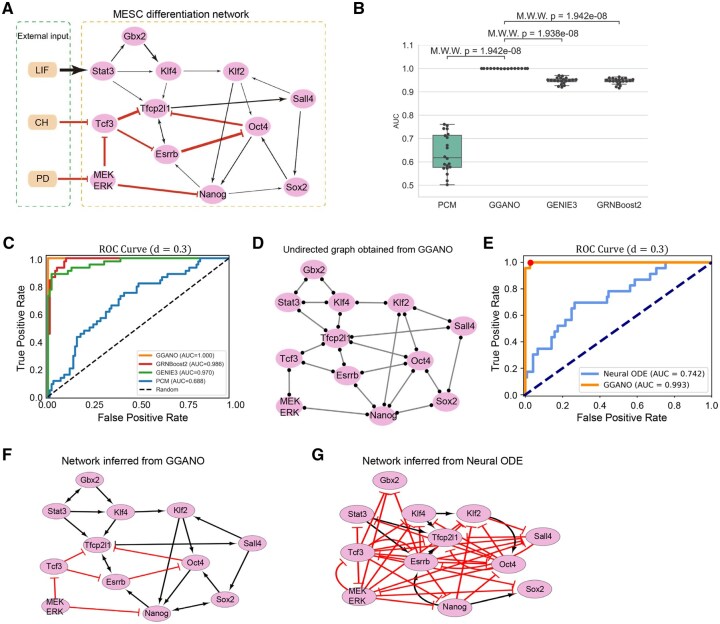
Comparison of network inference methods on MESC network. (A) Topology of the MESC network with 15 nodes and 26 interactions (18 activations and 8 inhibitions). Black arrows denote activation, red bars denote inhibition, orange nodes represent input signals, and pink nodes represent genes. (B) Boxplot of AUC values comparing GGANO, PCM, GENIE3, and GRNBoost2, showing the superior performance of GGANO. (C) ROC curves for GGANO, PCM, GENIE3, GRNBoost2, and a random baseline, further confirming GGANO’s advantage in network inference. (D) Undirected graph structure inferred by GGANO at noise level d=0.3. (E) ROC curves comparing pure Neural ODE and GGANO. (F) Final network structure of MESC inferred by GGANO under d=0.3. (G) Final network structure of MESC inferred by pure Neural ODE under d=0.3.

Next, we apply GGANO to several biologically realistic multistable networks. First, we examine the MESC differentiation network (Fig. 3A). Based on Eq. 1 in the Supplementary Material, we construct the corresponding mathematical formulation and introduce a constant $g_{0}=0.1$ as the basal synthesis rate. The parameters are set as a=0.3, b=0.2, S=0.5, n=4.0, and k=1.0, with all three external input variables fixed at 0.05.

At a high noise level of d=0.3, we compare the GGANO with existing network inference methods, including PCM (Leng *et al.* 2020), GENIE3 (Huynh-Thu *et al.* 2010), and GRNBoost2 (Moerman *et al.* 2019). We conduct 20 experiments, each with 10,000 sample points. Figure 3B shows the boxplot of AUC values for the four methods. It shows that GGANO outperforms the other methods, which is further supported by the Mann-Whitney-Wilcoxon (MWW) test, with p-values less than 0.001. We average the results from the 20 experiments and obtain the ROC curves for the four methods as well as the random method (Fig. 3C). These curves show that GGANO consistently outperforms the other methods across all experiments.

Figure 3D is the undirected network inferred by GGANO, which is in perfect agreement with the ground truth result (Fig. 3A). Next, we incorporate this undirected graph as prior knowledge into the training of the Neural ODE for network inference. By integrating this prior knowledge, the Neural ODE model exhibits a significant improvement in inference accuracy, with the AUC value increasing from 0.74 to 0.99 (Fig. 3E). Ultimately, we identify the structure associated with the point nearest to the upper left corner of the ROC curve (the red dot in Fig. 3E) as the inferred result of GGANO (Fig. 3F). This point maximizes the true positive rate while minimizing the false positive rate, thereby achieving optimal model performance. In contrast, the structure inferred by the pure Neural ODE method (Fig. 3G) deviates significantly from the ground truth result. The superior performance of GGANO is attributed to its ability to constrain the search space of the neural network by incorporating prior knowledge from the GGM, especially under high noise levels. This constraint reduces the impact of noisy data, guiding the model towards more accurate network inference. This conclusion is further validated by applying GGANO to a more complex network with additional feedback loops (Lang *et al.* 2021) (Supplementary Material: Section 7 and Fig. S2).

### Network inference for a human embryonic stem cell (HESC) network

**Figure 4.**
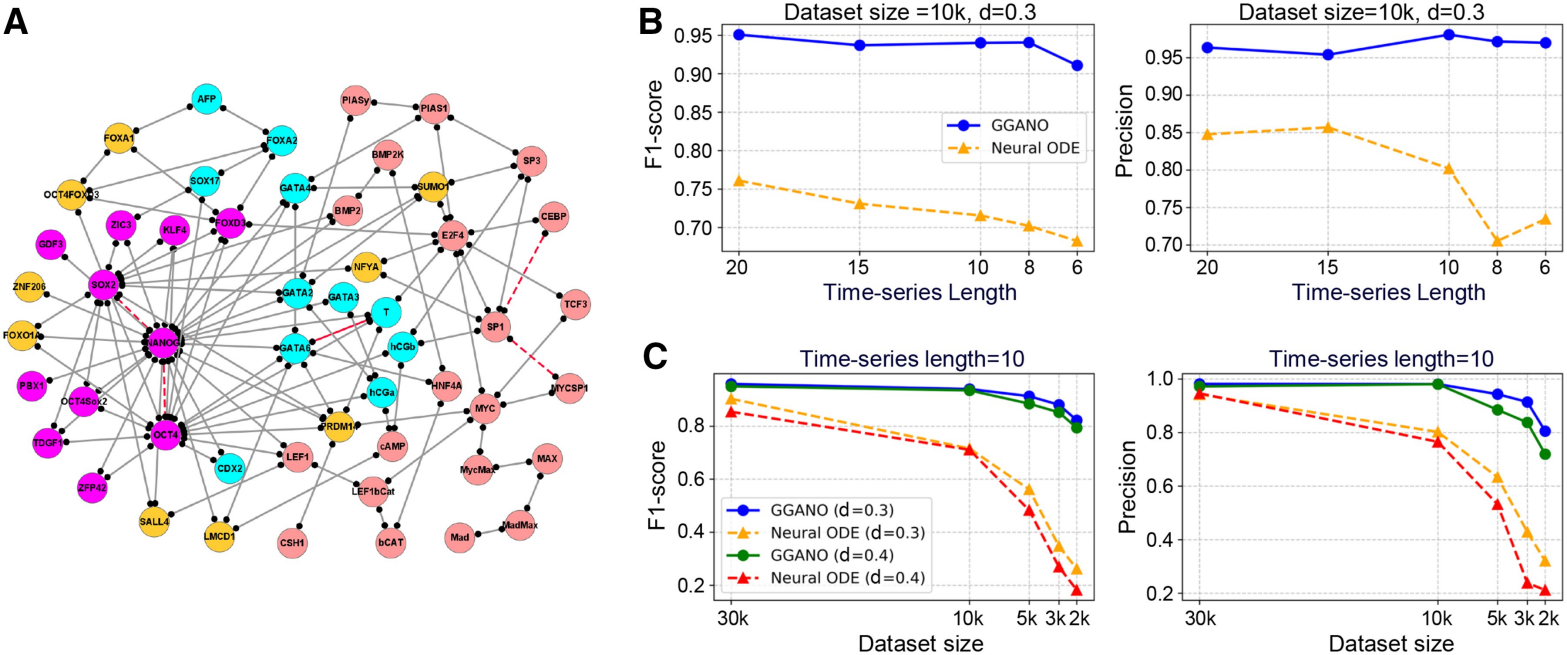
Impact of time-series length and dataset size on network inference performance. (A) The undirected graph structure of the HESC system inferred by GGANO when the noise level d=0.4. The gray solid lines represent the structure consistent with the reference network, while the red solid line indicates additional predictions, and the missing regulatory interactions are also marked by the red dotted lines. (B) Comparison of F1-score and Precision for the pure Neural ODE and GGANO as the time-series length (i.e. number of time points per trajectory) decreases, with a fixed training set size of 10,000 and a noise level of d=0.3. (C) Comparison of F1-score and Precision for the pure Neural ODE and GGANO as the training set size decreases, with a fixed time-series length of 10.

We further apply GGANO to a higher-dimensional HESC developmental network, whose topology is shown in Supplementary Material: Fig. S3. The dynamics of this network is determined by the mutual repression of major stem cell markers (NANOG, OCT4, SOX2) and major differentiation markers (GATA4, CDX2) (Boyer *et al.* 2005, Chickarmane and Peterson 2008, Niwa 2007). The parameters of the models are set as: a=0.37, b=0.5, S=0.5, n=3, k=1. Even under high noise (d=0.4), GGANO predicts 101 regulatory interactions, among which 100 are correct, while four interactions in the standard network are not recovered (Fig. 4A).

Next, we evaluate the influence of dataset size and time-series length on model performance using Precision and F1-score. When the training set is fixed at 10,000 samples under noise level d=0.3, the performance of the pure Neural ODE rapidly deteriorates as the number of time points decreases, whereas GGANO maintains high accuracy and robustness (Fig. 4B). Conversely, when the trajectory length is fixed at 10 or 20, reducing the training set size causes a sharp decline in the Neural ODE, highlighting its heavy reliance on large datasets in high-dimensional settings (Fig. 4C and Supplementary Material: Fig. S4). In contrast, GGANO achieves stable performance, with F1-scores around 0.8 even at 2,000 samples. These results underscore the superior robustness and practicality of GGANO for high-dimensional data with limited samples.

### Reconstructing networks from realistic single cell data

**Figure 5.**
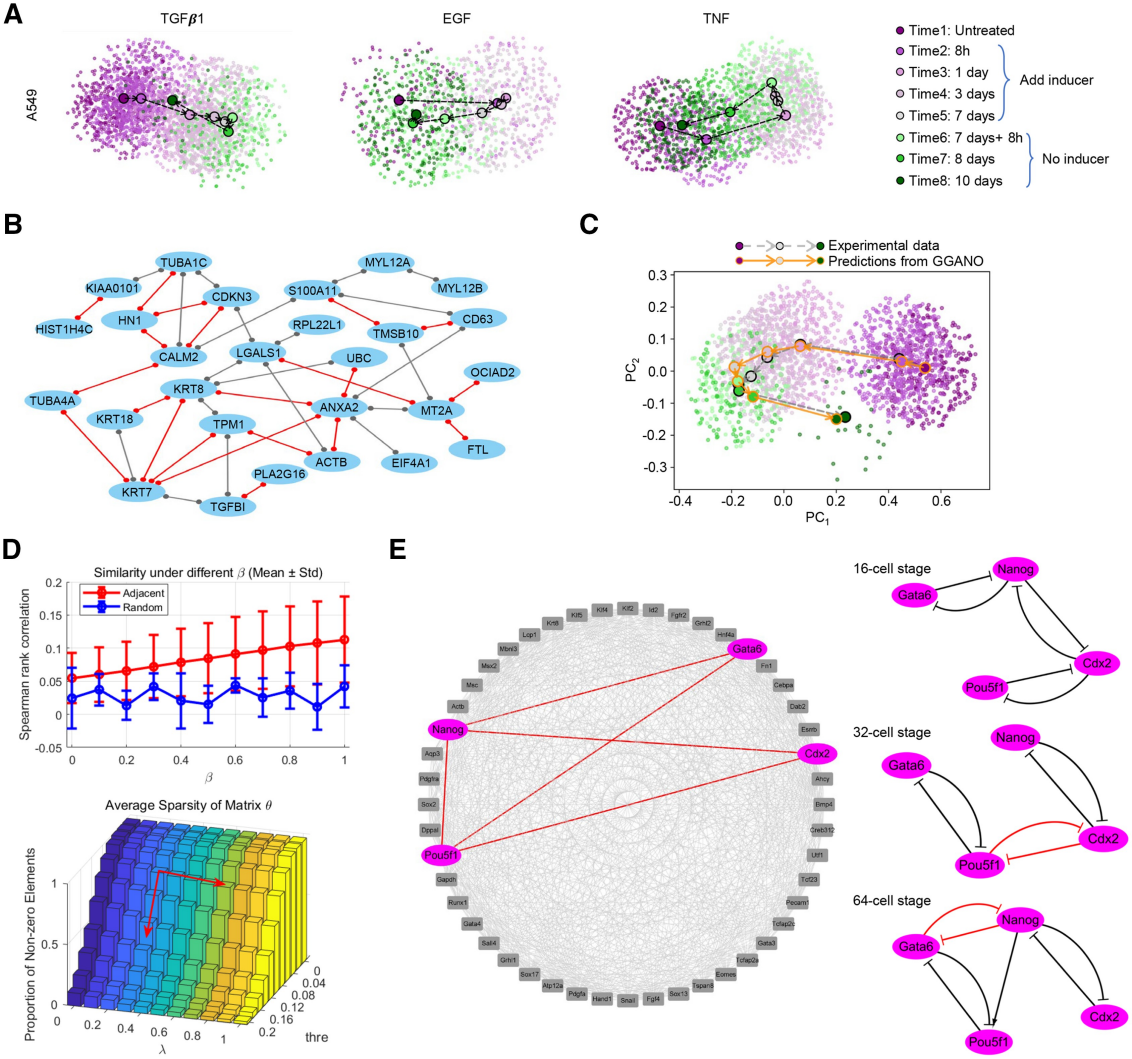
Application of GGANO to the single cell data. (A) PCA embeddings of the EMT time-course experiments performed in the A549 cell line with three different EMT-inducing factors. Each small point represents an individual sample, while larger points indicate the average of all data points at that time point. (B) Undirected network structure inferred by GGANO from A549 cell line data under TGFβ1 induction, consisting of 27 genes and 44 regulatory interactions. Red edges indicate interactions shared with networks inferred from A549 data under two other inducers. (C) GGANO fits the experimental data accurately using the structure in B) as prior knowledge. Orange curves denote GGANO predictions, and gray curves denote experimental observations. (D) Effects of varying hyperparameter settings in the GGANO on the inferred networks from the cell fate decision dataset. (E) The complete undirected network inferred by GGANO and the temporal changes of its key subnetwork.

To see how GGANO works on real-world data, we first analyze a scRNA-seq dataset of EMT time-course experiments (Cook and Vanderhyden 2020), focusing in particular on the A549 cell line, which exhibits the clearest EMT progression and the largest sample size (Fig. 5A, Supplementary Material: Fig. S6). Based on the scheme depicted in Fig. 1B, we can infer the time varying undirected graphs from the data involving a single inducer. However, when handling the data of diverse inducers, this might result in completely different results. Despite the fact that the binding of three distinct inducers to each cell surface receptor initiates independent signaling pathways, within the same cell line, we argue that the underlying regulatory network structure should possess a certain extent of similarity. Hence, we propose the scheme in Fig. 1C. Through solving the corresponding optimization problem (Materials and Methods), we can infer the regulatory networks under different inducers concurrently while preserving the structural similarity of these networks.

Since the distribution of sampling time points of the data is not uniform, we normalize the time, with $t_{1}=0$, $t_{2}=1/30$, $t_{3}=1/10$, $t_{4}=3/10$, $t_{5}=7/10$, $t_{6}=11/15$, $t_{7}=4/5$, $t_{8}=1$. Then, correspondingly, we can obtain $h_{2j}=t_{2}-t_{1}=1/30$, $h_{3j}=t_{3}-t_{2}=1/15$, $h_{4j}=1/5$, $h_{5j}=2/5$, $h_{6j}=1/30$, $h_{7j}=1/15$, $h_{8j}=1/5$, where j∈{1,2,3}. In accordance with the iterative formula Eq. 4, we can obtain the concentration matrix $\Theta_{ij}$. Here, i∈{1,2,…,8} represents the time points, and j∈{1,2,3} corresponds respectively to three different inducers (1: TGF β1; 2: EGF; 3: TNF). Considering the stability of biological processes, we assume that the underlying regulatory networks behind the EMT responses and their reversal processes triggered by different inducers are also stable. That is, for ∀j∈{1,2,3}, we have ${\bar{\Theta }}_{j}=\frac{1}{8}\sum_{i=1}^{8}\Theta_{ij}$. Through maximizing the likelihood function, we are able to determine the threshold to distinguish the zero elements and non-zero elements in the average concentration matrix ${\bar{\Theta }}_{j}$. Figure 5B presents the undirected graph structures inferred from the data with inducer TGF β1, which contains 44 regulations. While in the data of EGF and TNF, we infer 55 regulations (Supplementary Material: Fig. S7). And there are 21 common regulations in these three networks. Notably, when applying GGANO, we include 103 genes from the single-cell EMT datasets for the A549 cell line (treated with TGF β1, EGF, and TNF), and we retain 51 genes that are shared across these three inducing conditions. The network shown in Fig. 5B does not represent all retained genes; instead, it displays only the largest connected component after filtering. Specifically, nodes with degree zero and isolated two-node components (pairs connected only to each other) are removed, so that Fig. 5B highlights the primary regulatory structure inferred by GGANO.

Based on the structures depicted in Fig. 5B, we employ the Neural ODE to model the cell line influenced by different inducers separately. We add an extra node to the input layer of the neural network to signify the concentration of the inducer. The Neural ODE model succeeds in fitting the experimental data, which indirectly validates the reliability of the inferred undirected network. For instance, for the TGF β1 data, the predictions from GGANO (orange outlined circles) are in high agreement with the experimental data (black outlined circles), as depicted in Fig. 5C. The small points stand for individual sample point, while the large points indicate the mean of the corresponding small points. The purple-filled points signify the transition process from the epithelial state (E) to the mesenchymal state (M), whereas the green-filled points represent the reversal process. The results of the other two sets of data are shown in Supplementary Material: Fig. S8. Of note, the Neural ODE model not only fits the data at these eight time points, but also infers the state values of the cells between these time points.

As a second application for realistic data, we apply GGANO to scRNA-seq data from mouse pre-implantation development (Guo *et al.* 2010), aiming to capture temporal dynamics of regulatory networks (Fig. 5E and Supplementary Material: Fig. S5). This analysis uncovered interaction patterns consistent with established fate decisions and enabled a systematic evaluation of GGANO’s sensitivity to key hyperparameters (Fig. 5D and Supplementary Material: Section 8).

### Rebuilding the dynamics of EMT

**Figure 6.**
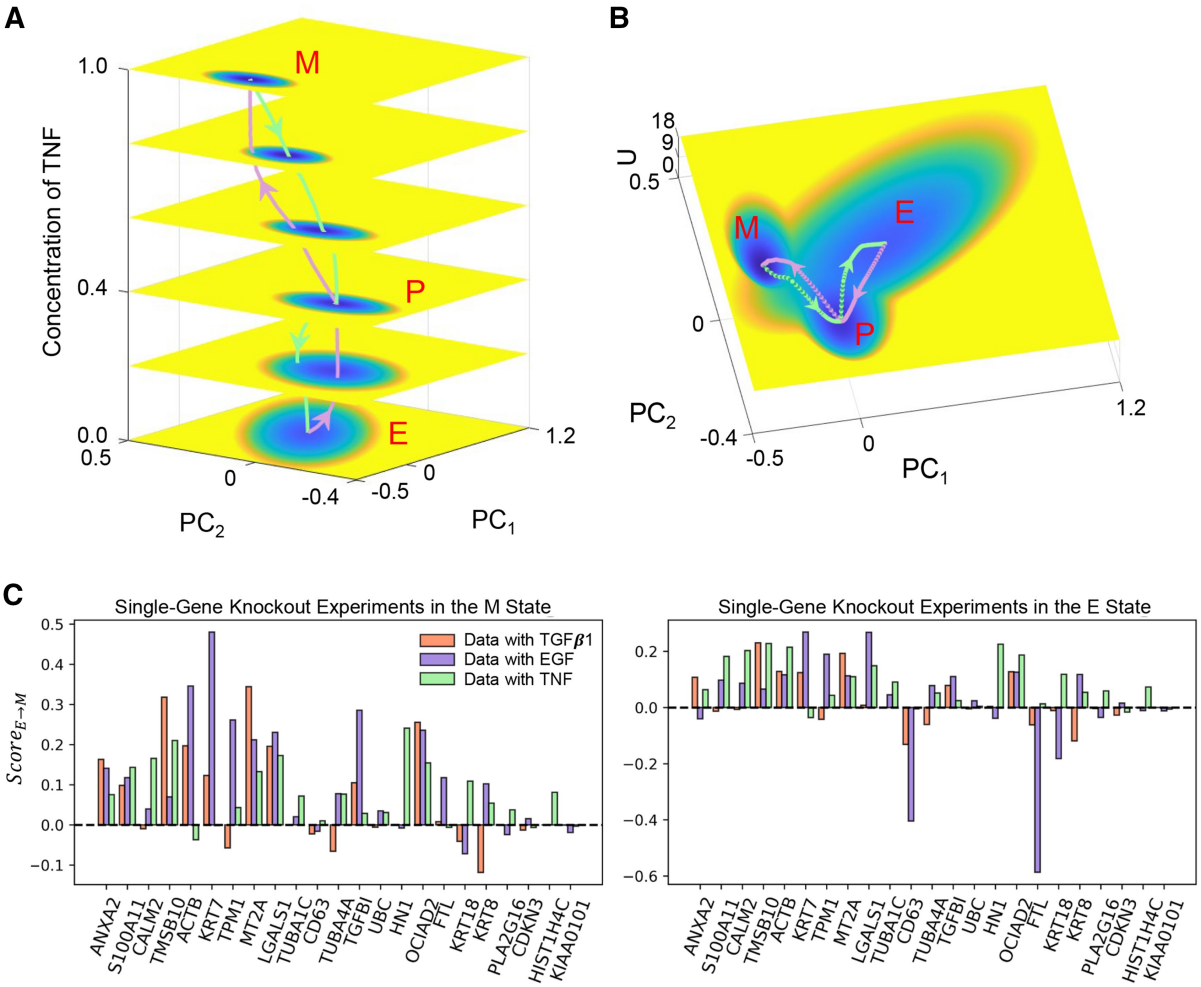
Potential landscape of TNF-Induced EMT. (A) The potential energy landscape of the system at different concentrations of the inducer TNF. When the concentration is 0.0, the system is in the E state, and as the concentration increases, the system gradually transitions from the E state to the M state. When the concentration reaches 1.0, the system is in the M state, and as the concentration decreases, the system returns from the M state to the E state. The purple line represents the transition path from the E state to the M state, while the green line represents the reverse transition from the M state to the E state. E: Epithelial state, M: Mesenchymal state, P: Partial EMT state. (B) The corresponding 3D potential energy landscape. (C) The calculation of $\mathrm{Scor}e_{E\to M}$ on three datasets from the A549 cell line. $\mathrm{Scor}e_{E\to M}>0$ indicates that the knockout of a specific gene drives the system away from the M state and toward a more epithelial-like state, suggesting that the gene may play an important role in the EMT process.

In addition to network inference, we further investigate the dynamic characteristics of EMT, including key genes involved in the transition from the E to M state, as well as the transition pathways between these two states. We first seek to explore the transition paths between E and M state. When we modify the concentration of the inducer in the Neural ODE model, the system exhibits various dynamic behaviors. As the value of the inducer gradually increases from 0.0 to 1.0, the system’s energy landscape shifts from the E to M state, with the transition path indicated by the purple line (Fig. 6A and Supplementary Material: Fig. S10). When the value of the inducer gradually decreases from 1.0 to 0.0, the system reverts from the M to E state with the transition path indicated by the green line. The purple line and the green line do not align, suggesting that the transition path is irreversible, which arises from the nonzero flux in the non-equilibrium system (Chen *et al.* 2023, Li and Wang 2014).

We discover that for the transition between the E and M state, the system passes through an intermediate state (Fig. 6B and Supplementary Material: Fig. S10). This state lies between the E and M state, presenting a transitional feature, neither fully retaining epithelial properties nor completely acquiring mesenchymal properties (also referred to as the partial EMT state or hybrid EMT state). Specifically, taking the TNF data as an instance, we carry out the analysis for gene expression. For cells in the M state, the M markers (such as TMSB10, ACTB, KRT7, TPM1, MT2A, LGALS1, TGFBI, and OCIAD2) exhibit markedly high expression features, while the E marker genes (such as KRT18, PLA2G16, and HIST1H4C) demonstrate low expression. Correspondingly, cells in the E state present the opposite expression characteristics. For cells in the intermediate state, these markers are expressed to a middle degree.

Through the energy landscape and the transition path, we discover that neither the EMT nor its reverse process is a binary one but progresses step by step. The system initially shifts to the partial EMT state, and then based on environmental variations, it will further transform into other states. For instance, when the concentration of the inducer rises, the system first transforms from the E state to the partial EMT state. If no additional inducer is supplemented in the subsequent period, the concentration of the inducer will gradually decline, and the system will revert from the partial EMT state to the E state. On the contrary, if the concentration of the inducer keeps increasing, the system will further go to the M state. Consistent with these dynamics, our results demonstrate that GGANO successfully captures regulatory features specific to the partial EMT state, underscoring its role as a distinct and highly plastic intermediate state. Moreover, accumulating evidence suggests that the partial EMT state enhances stemness, immune evasion, and chemoresistance, thereby indirectly facilitating tumor metastasis (Jiang and Zhan 2020, Li *et al.* 2023, Wilson *et al.* 2020). Consequently, preventing cells from transitioning into the partial EMT state may represent a novel avenue for suppressing tumor initiation, progression, and metastasis, while therapeutic strategies targeting this state could also provide a foundation for personalized treatment.

Next, we aim to identify the key genes involved in EMT. To achieve this, we conduct single-gene knockout experiments on the Neural ODE model. By analyzing the variations in the stable state of the system before and after the knockout experiment, we can infer the potential role of the gene in the transition process. Specifically, in the input layer of the Neural ODE, we set the node value representing the concentration of the inducer to 1.0 to simulate the transition process from the E state to the M state, and define the resulting stable state as the M state, denoted as $x_{ss}^{M}$. Conversely, to simulate the reversal process, we set the node value to 0.0 and define the stable state as the E state, represented by $x_{ss}^{E}$. The M state generated by our model is highly consistent with the data at the fifth time point across the three datasets. However, for the TGFβ1 and TNF data, some deviation exists between the E state generated by GGANO and the corresponding experimental data at the first and eighth time points (Supplementary Material: Fig. S9). This discrepancy may be attributed to the fact that the data are only observed for three days after the withdrawal of the inducer, and some cells may not have fully completed the reversal process from the M state to the E state.

To quantify the functional importance of individual genes in regulating EMT, we introduced an indicator, $\mathrm{Scor}e_{E\to M}$, which characterizes the extent to which gene knockouts affect the EMT. A positive value of $\mathrm{Scor}e_{E\to M}$ (see detailed definitions and computational procedures in Supplementary Material: Section 6) suggests that the gene facilitates this transition, with larger values indicating a stronger effect. From the calculation of $\mathrm{Scor}e_{E\to M}$ on the three datasets, we find that in the knockout experiments of both the M and E states, the $\mathrm{Scor}e_{E\to M}$ of genes TMSB10, MT2A, LGALS1, TGFBI, and OCIAD2 were all positive (Fig. 6C). Importantly, these five key genes we predicted to facilitate the EMT are supported by previous literature (Supplementary Material: Section 9), suggesting that they may serve as significant biomarkers and potential targets for suppressing EMT.

## Discussion

Regulatory redundancy is a fundamental feature of biological systems, ensuring that alternative pathways can compensate when one route is disrupted. In this context, accurately inferring the structure of gene regulatory networks is particularly challenging, as high dimensionality and noise often yield multiple plausible solutions. Our framework, GGANO, addresses this issue by integrating GGM with Neural ODE, enabling prior structural information to constrain network inference. This hybrid strategy not only enhances sparsity and interpretability, but also reduces the parameter search space and dependence on long trajectories. Application of GGANO to EMT-related scRNA-seq datasets demonstrates its ability to recover robust regulatory patterns across conditions. Furthermore, by identifying genes associated with the EMT and linking them to landscape-based analyses, our results highlight the role of intermediate cell states with high plasticity in driving cell fate transitions.

To further assess practicality, we benchmark the computational efficiency of GGANO on a single-cell dataset with 48 variables and 60,000 trajectories, each of length 30. On average, estimating the prior graph with GGM requires approximately 10 seconds. Training a standard Neural ODE without prior constraints takes about 20 seconds per epoch. By contrast, when prior constraints from GGM are incorporated (Fig. 1D), the training cost rises substantially, since a separate Neural ODE must be executed for each variable. Under this setting, the average training time reaches nearly 10 minutes per epoch. However, this longer training also provides greater flexibility as different prior structural constraints can be introduced at each time point. This setting reflects the fact that certain gene regulatory interactions may occur only during specific temporal periods, thereby yielding a more accurate model.

From an application perspective, the integration of GGM priors effectively reduces GGANO’s reliance on long temporal trajectories, enabling robust network inference even when time-series lengths are moderately shortened. However, datasets with fewer than five temporal measurements remain challenging for this framework. Such cases may be partially addressed using pseudotime ordering or other trajectory reconstruction methods to enrich temporal resolution. Moreover, while GGANO performs well in moderate dimensions, scaling to ultra–high-dimensional systems with thousands of variables remains computationally demanding. In such scenarios, focusing on core subnetworks of interest, as demonstrated in this study, provides a practical compromise. While this strategy inevitably omits certain global interactions and may introduce some bias, it nonetheless represents a feasible and effective approach for extracting mechanistic insights from large-scale biological systems.

In summary, GGANO developed in this work furnishes a general computational framework for inferring the structure of gene regulatory networks and investigating the stochastic dynamics of high-dimensional systems. Our findings advance the understanding of the EMT process and the mechanism of cell fate transitions.

## Supplementary Material

btaf598_Supplementary_Data

## Data Availability

The TaqMan low-density array transcription factor data for cell fate decision studies reported by Guo *et al.* (2010) can be accessed from the Mouse Genome Informatics database under accession number J: 140465. The EMT single-cell RNA-seq data reported by Cook and Vanderhyden (2020) can be accessed from the NCBI Gene Expression Omnibus under accession number GSE147405.
