# Supplementary material for: Reconstructing gene network structure and dynamics from single cell data
Source: Bioinformatics. 2025 Nov 3;41(11):btaf598. doi: 10.1093/bioinformatics/btaf598 (PMC12603361; doi:10.1093/bioinformatics/btaf598)
Supplement: btaf598_Supplementary_Data [file btaf598_supplementary_data.pdf]

Supplementary Information for

Reconstructing gene network structure and dynamics from single cell data

Feng Chen, Chunhe Li

Chunhe Li.

E-mail: chunheli@fudan.edu.cn

This PDF file includes:

Supplementary text

Figs S1 to S10

SI References

Supporting Information Text

1. EMT data preprocessing

For each of the 12 conditions, samples were collected at the initial time point, four distinct time points ranging from 8 hours to 7 days after the treatment, and three additional time points ranging from 8 hours to 3 days after the EMT-inducing stimulus was removed. The resulting dataset includes 3,568 cells and 13,239 genes under TGFβ1 induction, 12,911 cells and 13,143 genes under TNF induction, and 12,435 cells with 13,132 genes under EGF induction. To concentrate on biologically meaningful signatures, we restrict the analysis to the 103 EMT-associated genes reported in (1). This curated set was defined as all genes differentially expressed in at least eight of the twelve conditions, corresponding to two-thirds of the experiments, thereby achieving a balance between sensitivity and specificity.

Unique Molecular Identifier (UMI) counts are processed into normalized gene expression values through a standard workflow. For each cell, raw UMI counts are first divided by the total UMI count and scaled by a constant factor (e.g., 10,000) to correct for library size differences. The scaled values are then log-transformed to stabilize variance and yield comparable distributions across genes, providing relative expression levels. Genes with more than 50% missing values are first removed. For the remaining genes, missing or near-zero expression values are imputed using k-nearest neighbors (KNN), where each missing entry is inferred from the five most similar cells in expression space. This procedure mitigates the impact of dropout events and enhances data robustness. After imputation, gene expression values are normalized by subtracting the minimum and dividing by the range (maximum minus minimum) for each gene, yielding relative expression levels between 0 and 1.

2. Mathematical model of gene regulatory networks for generating simulation data

For these gene regulatory networks in this article, Hill functions are often used to describe the activation or inhibition regulations (2–4):

$$\begin{aligned}\frac{dx_i}{dt} &= \sum_{j=1}^{N} \frac{A_{ji}\times x_j^n}{S_{ji}^n+x_j^n} + \sum_{j=1}^{N} \frac{B_{ji}\times S_{ji}^n}{S_{ji}^n+x_j^n} - k_i \times x_i + \xi_i \\ &= f_i(\boldsymbol{x}) + \xi_i.\end{aligned} \quad [1]$$

Here, where f_i is the driving force of the system, $x_i (i = 1, 2, ..., N)$ represents the expression level of gene X_i, k represents the basal degradation rate of X_i, S_{ji} represents the threshold of a sigmoidal function, and n is the Hill coefficient, which determines the steepness of the sigmoidal function. $\boldsymbol{\xi}$ is the Gaussian white noise, whose autocorrelation function is $\langle \xi_i(\boldsymbol{x}, t), \xi_j(\boldsymbol{x}, 0)\rangle = 2d\,\delta(t)$, and d is the diffusion coefficient. Besides, A and B are respectively the interaction matrix for the activation and inhibition. A_{ji} measures the strength of the activation of X_j on X_i, and when $A_{ji} = 0$, it means that there is no activation from component X_j to component X_i. Similarly, B_{ji} measures the strength of the inhibition of X_j on X_i, and when $B_{ji} = 0$, it means that there is no inhibition from component X_j to component X_i. Then, we can obtain the iterative formula for Eq. (1):

$$x_i(t + \Delta t) = x_i(t) + f_i(\boldsymbol{x}(t))\Delta t + \sqrt{2d\Delta t}\eta, \quad i = 1, 2, \ldots, N, \quad [2]$$

where η is a standard normal random variable with zero mean and unit variance. This formulation allows the generation of simulated time-series data corresponding to the underlying regulatory networks. In our simulations, we typically set the step size between 0.01 and 0.1 and generated time series data under different noise levels. To construct datasets, we sampled random initial conditions and selected subsequent 10-30 time points to form each sequence.

3. Gaussian graph model

A. Definition and properties.

Definition 1 (Undirected Gaussian Graph Model)**.** *$G = (V, E)$ is an undirected graph, where $V = (1, 2, ..., p)$ is the set of nodes and E is the set of edges. A random vector $X \in \mathbb{R}^p$ is named to satisfy the undirected Gaussian graphical model with graph G if X follows a multivariate Gaussian distribution $\mathcal{N}(\mu, \Sigma)$ and satisfies*

$$\left(\Sigma^{-1}\right)_{i,j} = 0, \quad \textit{for all } (i, j) \notin E.$$

Assume that the random vector $X \in \mathbb{R}^p$ follows a multivariate Gaussian distribution $\mathcal{N}(\mu, \Sigma)$, with parameters $\mu \in \mathbb{R}^p$ (mean) and $\Sigma \in \mathbb{S}^p_{\succ 0}$ (covariance matrix, $\mathbb{S}^p_{\succ 0}$ is the set of all symmetric positive definite matrixs of size $N \times N$). Let Θ denote the inverse of the covariance matrix, i.e., $\Theta = \Sigma^{-1}$, which is also referred to as the precision matrix or concentration matrix. The density function can be expressed as:

$$f_{\mu,\Theta}(x) = \exp\left\{\mu^T \Theta x - \left\langle \Theta, \frac{1}{2} x x^T \right\rangle - \frac{p}{2}\log(2\pi) + \frac{1}{2}\log\det(\Theta) - \frac{1}{2}\mu^T \Theta \mu\right\}, \qquad [3]$$

where $< \cdot, \cdot >$ represents the inner product. For disjoint subsets A, B and C in V, $X_A \perp\!\!\!\perp X_B \mid X_C$ denotes that X_A and X_B are conditionally independent given X_C. If $C = \emptyset$, it can be simplified to $X_A \perp\!\!\!\perp X_B$.

Corollary 1. *Let the random vector $X \in \mathbb{R}^p$ follow the distribution $\mathcal{N}(\mu, \Sigma)$. Suppose $i, j \in V$ and $i \neq j$, then we have:*
(i) $X_i \perp\!\!\!\perp X_j$ if and only if $\Sigma_{i,j} = 0$,
(ii) $X_i \perp\!\!\!\perp X_j \mid X_{V \setminus \{i,j\}}$ if and only if $\Theta_{i,j} = 0$, if and only if $\det\left(\Sigma_{V\setminus\{i\}, V\setminus\{j\}}\right) = 0$.

Proof. (i) Divide X into two parts $X_1 \in \mathbb{R}^a$ and $X_2 \in \mathbb{R}^b$, such that $a + b = p$. Similarly, partition μ and Σ as follows:

$$\mu = \begin{pmatrix} \mu_1 \\ \mu_2 \end{pmatrix}, \quad \Sigma = \begin{pmatrix} \Sigma_{11} & \Sigma_{12} \\ \Sigma_{21} & \Sigma_{22} \end{pmatrix},$$

where Σ_{22} is a positive-definite matrix. Consider the following linear system of equations:

$$\begin{pmatrix} \Sigma_{11} & \Sigma_{12} \\ \Sigma_{21} & \Sigma_{22} \end{pmatrix} \begin{pmatrix} x \\ y \end{pmatrix} = \begin{pmatrix} c \\ d \end{pmatrix}.$$

Since Σ_{22} is invertible, we solve the second equation to obtain:

$$y = \Sigma_{22}^{-1}(d - \Sigma_{21}x),$$

substituting y into the first equation:

$$\Sigma_{11}x + \Sigma_{12}(\Sigma_{22}^{-1}(d - \Sigma_{21}x)) = c,$$

which simplifies to $(\Sigma_{11} - \Sigma_{12}\Sigma_{22}^{-1}\Sigma_{21})x = c - \Sigma_{12}\Sigma_{22}^{-1}d$. Since the Schur complement of Σ_{22} in Σ is invertible, we can have

$$\begin{aligned} x &= (\Sigma_{11} - \Sigma_{12}\Sigma_{22}^{-1}\Sigma_{21})^{-1}(c - \Sigma_{12}\Sigma_{22}^{-1}d) \\ &= (\Sigma_{11} - \Sigma_{12}\Sigma_{22}^{-1}\Sigma_{21})^{-1}c - (\Sigma_{11} - \Sigma_{12}\Sigma_{22}^{-1}\Sigma_{21})^{-1}\Sigma_{12}\Sigma_{22}^{-1}d. \end{aligned}$$

Substituting x into the expression for y, we obtain:

$$\begin{aligned} y &= \Sigma_{22}^{-1}(d - \Sigma_{21}(\Sigma_{11} - \Sigma_{12}\Sigma_{22}^{-1}\Sigma_{21})^{-1}(c - \Sigma_{12}\Sigma_{22}^{-1}d)) \\ &= -\Sigma_{22}^{-1}\Sigma_{21}(\Sigma_{11} - \Sigma_{12}\Sigma_{22}^{-1}\Sigma_{21})^{-1}c + (\Sigma_{22}^{-1} + \Sigma_{22}^{-1}\Sigma_{21}(\Sigma_{11} - \Sigma_{12}\Sigma_{22}^{-1}\Sigma_{21})^{-1}\Sigma_{12}\Sigma_{22}^{-1})d. \end{aligned}$$

Thus, the inverse of Σ can be expressed as:

$$\begin{aligned} \Sigma^{-1} &= \begin{pmatrix} \Sigma_{11} & \Sigma_{12} \\ \Sigma_{21} & \Sigma_{22} \end{pmatrix}^{-1} = \begin{pmatrix} \Sigma_{11\cdot 2}^{-1} & -\Sigma_{11\cdot 2}^{-1}\Sigma_{12}\Sigma_{22}^{-1} \\ -\Sigma_{22}^{-1}\Sigma_{21}\Sigma_{11\cdot 2}^{-1} & \Sigma_{22}^{-1} + \Sigma_{22}^{-1}\Sigma_{21}\Sigma_{11\cdot 2}^{-1}\Sigma_{12}\Sigma_{22}^{-1} \end{pmatrix} \\ &\triangleq \begin{pmatrix} \Sigma_{(1,1)}^{-1} & \Sigma_{(1,2)}^{-1} \\ \Sigma_{(2,1)}^{-1} & \Sigma_{(2,2)}^{-1} \end{pmatrix}, \end{aligned}$$

where $\mathbf{\Sigma}_{11\cdot 2} = \mathbf{\Sigma}_{11} - \mathbf{\Sigma}_{12}\mathbf{\Sigma}_{22}^{-1}\Sigma_{21}$. The density function of X_2 can then be written as:

$$f\left(\boldsymbol{x}_2\right) = \frac{1}{(2\pi)^{\frac{b}{2}}\left|\Sigma_{22}\right|^{\frac{1}{2}}} e^{-\frac{1}{2}(x_2-\mu_2)^T\Sigma_{22}^{-1}(x_2-\mu_2)},$$

given $\boldsymbol{X}_2 = \boldsymbol{x}_2$, the conditional density function of X_1 is

$$\begin{aligned} f\left(\boldsymbol{x_1} \mid \boldsymbol{x_2}\right) &= \frac{f(\boldsymbol{x})}{f\left(\boldsymbol{x}_2\right)} \\ &= \frac{1}{(2\pi)^{\frac{a}{2}}\left|\Sigma_{11\cdot 2}\right|^{\frac{1}{2}}} e^{-\frac{1}{2}\left\{\Sigma_{i=1}^2\Sigma_{j=1}^2(\boldsymbol{x}_i-\mu_i)^T\Sigma_{(i,j)}^{-1}(\boldsymbol{x}_j-\mu_j)-(x_2-\mu_2)^T\Sigma_{22}^{-1}(x_2-\mu_2)\right\}}. \end{aligned}$$

The expression in the exponent of e can be simplified as:

$$\begin{aligned} &\sum_{i=1}^{2}\sum_{j=1}^{2}\left(\boldsymbol{x}_i-\boldsymbol{\mu}_i\right)^T\Sigma_{(i,j)}^{-1}\left(\boldsymbol{x}_j-\boldsymbol{\mu}_j\right) - \left(\boldsymbol{x}_2-\boldsymbol{\mu}_2\right)^T\Sigma_{22}^{-1}\left(\boldsymbol{x}_2-\boldsymbol{\mu}_2\right) \\ &= \left(\boldsymbol{x}_1-\boldsymbol{\mu}_1\right)^T\Sigma_{(1,1)}^{-1}\left(\boldsymbol{x}_1-\boldsymbol{\mu}_1\right) + \left(\boldsymbol{x}_1-\boldsymbol{\mu}_1\right)^T\Sigma_{(1,2)}^{-1}\left(\boldsymbol{x}_2-\boldsymbol{\mu}_2\right) + \left(\boldsymbol{x}_2-\boldsymbol{\mu}_2\right)^T\Sigma_{(2,1)}^{-1}\left(\boldsymbol{x}_1-\boldsymbol{\mu}_1\right) \\ &\qquad + \left(\boldsymbol{x}_2-\boldsymbol{\mu}_2\right)^T\left(\mathbf{\Sigma}_{(2,2)}^{-1}-\mathbf{\Sigma}_{22}^{-1}\right)\left(\boldsymbol{x}_2-\boldsymbol{\mu}_2\right) \\ &= \left[\left(\boldsymbol{x}_1-\boldsymbol{\mu}_1\right)^T\Sigma_{11\cdot 2}^{-1}\left(\boldsymbol{x}_1-\boldsymbol{\mu}_1\right) - \left(\boldsymbol{x}_1-\boldsymbol{\mu}_1\right)^T\Sigma_{11\cdot 2}^{-1}\Sigma_{12}\Sigma_{22}^{-1}\left(\boldsymbol{x}_2-\boldsymbol{\mu}_2\right)\right] \\ &\qquad - \left(\boldsymbol{x}_2-\boldsymbol{\mu}_2\right)^T\Sigma_{22}^{-1}\Sigma_{21}\Sigma_{11\cdot 2}^{-1}\left(\boldsymbol{x}_1-\boldsymbol{\mu}_1\right) + \left(\boldsymbol{x}_2-\boldsymbol{\mu}_2\right)^T\Sigma_{22}^{-1}\Sigma_{21}\Sigma_{11\cdot 2}^{-1}\Sigma_{12}\Sigma_{22}^{-1}\left(\boldsymbol{x}_2-\boldsymbol{\mu}_2\right) \\ &= \left(\boldsymbol{x}_1-\boldsymbol{\mu}_1\right)^T\Sigma_{11\cdot 2}^{-1}\left[\left(\boldsymbol{x}_1-\boldsymbol{\mu}_1\right) - \Sigma_{12}\Sigma_{22}^{-1}\left(\boldsymbol{x}_2-\boldsymbol{\mu}_2\right)\right] \\ &\qquad + \left(\boldsymbol{x}_2-\boldsymbol{\mu}_2\right)^T\Sigma_{22}^{-1}\Sigma_{21}\Sigma_{11\cdot 2}^{-1}\left[\Sigma_{12}\Sigma_{22}^{-1}\left(\boldsymbol{x}_2-\boldsymbol{\mu}_2\right) - \left(\boldsymbol{x}_1-\boldsymbol{\mu}_1\right)\right] \\ &= \left[\left(\boldsymbol{x}_1-\boldsymbol{\mu}_1\right)^T - \left(\boldsymbol{x}_2-\boldsymbol{\mu}_2\right)^T\Sigma_{22}^{-1}\Sigma_{21}\right]\Sigma_{11\cdot 2}^{-1}\left[\left(\boldsymbol{x}_1-\boldsymbol{\mu}_1\right) - \Sigma_{12}\Sigma_{22}^{-1}\left(\boldsymbol{x}_2-\boldsymbol{\mu}_2\right)\right] \\ &= \left[\left(\boldsymbol{x}_1-\boldsymbol{\mu}_1\right) - \Sigma_{12}\Sigma_{22}^{-1}\left(\boldsymbol{x}_2-\boldsymbol{\mu}_2\right)\right]^T\Sigma_{11\cdot 2}^{-1}\left[\left(\boldsymbol{x}_1-\boldsymbol{\mu}_1\right) - \Sigma_{12}\Sigma_{22}^{-1}\left(\boldsymbol{x}_2-\boldsymbol{\mu}_2\right)\right]. \end{aligned}$$

It can be observed that $f\left(X_1 \mid X_2 = x_2\right)$ remains a normal distribution, with the mean and variance given as:

$$\begin{aligned} \mu_{1|2} &= \boldsymbol{\mu}_1 + \Sigma_{12}\Sigma_{22}^{-1}\left(\boldsymbol{x}_2-\boldsymbol{\mu}_2\right), \\ \Sigma_{1|2} &= \mathbf{\Sigma}_{11\cdot 2}^{-1} = \Sigma_{11} - \Sigma_{12}\mathbf{\Sigma}_{22}^{-1}\Sigma_{21}. \end{aligned} \qquad [4]$$

From Equation Eq. (4), it follows that $X_i \perp\!\!\!\perp X_j$ if and only if $\Sigma_{i,j} = 0$.

(ii) If $X_i \perp\!\!\!\perp X_j \mid X_{[V]\setminus\{i,j\}}$, it is equivalent to the 2×2 conditional covariance matrix being diagonal. From the conditional covariance in Equation Eq. (4), this is equivalent to $\Sigma_{\{i,j\},\{i,j\}}^{-1}$ being diagonal,

which implies that $\Theta_{\{i,j\},\{i,j\}}$ is diagonal, and this is further equivalent to $\Theta_{i,j} = 0$. The second equivalence follows from the cofactor formula for matrix inversion. For a general invertible matrix A, we have $A^{-1} = \frac{A^*}{det(A)}$, where A^* is the adjugate matrix of A, $A^*_{ij} = (-1)^{i+j} M_{ji}$, and M_{ji} is the minor of A obtained by removing the j-th row and i-th column. Thus, we can conclude:

$$\Theta_{ji} = \Sigma_{ji}^{-1} = (-1)^{i+j} \frac{\det\left(\Sigma_{[V]\setminus\{i\},[V]\setminus\{j\}}\right)}{\det(\Sigma)}.$$

□

By Corollary 1, zeros in the precision matrix correspond to conditional independence. Therefore, inferring the structure of the network is equivalent to identifying the zeros in the precision matrix.

B. Estimation of the Precision Matrix. Independently sampling n observations $X^{(1)}, X^{(2)}, ..., X^{(n)}$ from the distribution $\mathcal{N}(\mu, \Sigma)$, the sample mean and sample covariance matrix can be computed as:

$$\bar{X} = \frac{1}{n}\sum_{i=1}^{n} X^{(i)}, \quad S = \frac{1}{n}\sum_{i=1}^{n}(X^i - \bar{X})(X^i - \bar{X})^T,$$

and the likelihood function for these n samples can be written down as

$$\begin{aligned}&\prod_{i=1}^{n} \frac{1}{(2\pi)^{p/2}(\det\Sigma)^{1/2}} \exp\left\{-\frac{1}{2}(X^{(i)} - \mu)^T\Sigma^{-1}(X^{(i)} - \mu)\right\} \\ &= \frac{1}{(2\pi)^{np/2}(\det\Sigma)^{n/2}} \exp\left\{-\frac{1}{2}\sum_{i=1}^{n}(X^{(i)} - \mu)^T\Sigma^{-1}(X^{(i)} - \mu)\right\}.\end{aligned} \qquad [5]$$

Take the logarithm of equation Eq. (5) and simplify:

$$
\begin{aligned}
&-\frac{np}{2}log(2\pi)-\frac{n}{2}log(\det\Sigma)-\frac{1}{2}\sum_{i=1}^{n}(X^{(i)}-\mu)^T\Sigma^{-1}(X^{(i)}-\mu)\\
&=-\frac{np}{2}log(2\pi)-\frac{n}{2}log(\det\Sigma)-\frac{1}{2}\sum_{i=1}^{n}[(X^{(i)}-\bar{X})+(\bar{X}-\mu)]^T\Sigma^{-1}[(X^{(i)}-\bar{X})+(\bar{X}-\mu)]\\
&=-\frac{np}{2}log(2\pi)-\frac{n}{2}log(\det\Sigma)-\frac{1}{2}\sum_{i=1}^{n}[(X^{(i)}-\bar{X})^T\Sigma^{-1}(X^{(i)}-\bar{X})+(X^{(i)}-\bar{X})^T\Sigma^{-1}(\bar{X}-\mu)\\
&\qquad+(\bar{X}-\mu)^T\Sigma^{-1}(X^{(i)}-\bar{X})+(\bar{X}-\mu)^T\Sigma^{-1}(\bar{X}-\mu)]\\
&=-\frac{np}{2}log(2\pi)-\frac{n}{2}log(\det\Sigma)-\frac{1}{2}\sum_{i=1}^{n}[(X^{(i)}-\bar{X})^T\Sigma^{-1}(X^{(i)}-\bar{X})+(\bar{X}-\mu)^T\Sigma^{-1}(\bar{X}-\mu)]\\
&=-\frac{np}{2}log(2\pi)-\frac{n}{2}log(\det\Sigma)-\frac{1}{2}tr((\sum_{i=1}^{n}(X^{(i)}-\bar{X})(X^{(i)}-\bar{X})^T)\Sigma^{-1})-\frac{n}{2}(\bar{X}-\mu)^T\Sigma^{-1}(\bar{X}-\mu)\\
&=-\frac{np}{2}log(2\pi)-\frac{n}{2}log(\det\Sigma)-\frac{n}{2}tr(S\Sigma^{-1})-\frac{n}{2}(\bar{X}-\mu)^T\Sigma^{-1}(\bar{X}-\mu).
\end{aligned}
$$

In the case where $\hat{\mu}=\bar{X}$, the problem of maximizing the log-likelihood for the covariance matrix Σ reduces to the following optimization problem:

$$
\begin{aligned}
\max_{\Sigma}\quad & -\log\det(\Sigma)-\mathrm{tr}(S\Sigma^{-1}),\\
\text{s.t.}\quad & \Sigma\in\mathbb{S}^p_{\succ 0}.
\end{aligned}
$$

Becasuse of $\Theta=\Sigma^{-1}$, the optimized problem can be written as:

$$
\begin{aligned}
\max_{\Theta}\quad & \log\det(\Theta)-\mathrm{tr}(S\Theta),\\
\text{s.t.}\quad & \Theta\in\mathbb{S}^p_{\succ 0}.
\end{aligned}
\qquad [6]
$$

This implies that the maximum likelihood estimation for a Gaussian graphical model is a convex optimization problem. In general, we aim to learn a concise and interpretable graph structure from the data, especially for high-dimensional networks. This requires solving for a sparse concentration matrix, i.e., a concentration matrix with many zero elements, from problem Eq. (6). To achieve this, the Lasso regularization (L1 regularization) is introduced to constrain the off-diagonal elements of

the concentration matrix:

$$\begin{aligned} \min_{\Theta} \quad & -\log\det(\Theta) + \mathrm{tr}(S\Theta) + \lambda\|\Theta\|_{od,1}, \\ \text{s.t.} \quad & \Theta \in \mathbb{S}^{p}_{\succ 0}, \end{aligned} \qquad [7]$$

where $\|\Theta\|_{od,1}$ represents the semi-norm of Θ, defined as $\|\Theta\|_{od,1} = \sum_{i \neq j} |\Theta_{ij}|$. $\lambda \geq 0$ is the parameter to control the sparsity, with larger values of λ leading to sparser Θ. Additionally, Lasso regularization helps prevent overfitting by imposing a constraint on the model's complexity during the fitting process.

C. Multi-conditional Temporal Gaussian Graphical Model. Biological systems often exhibit stability, allowing us to assume that intracellular regulatory networks have a stable structure, meaning the network structure does not change drastically over short periods. We extend the optimization problem in Eq. (7) by incorporating the similarity of network structures at adjacent time points as a new constraint:

$$\begin{aligned} \min_{\Theta_i} \quad & \sum_{i=1}^{T} \left[-n_i \left(\log\det\Theta_i - \mathrm{tr}\left(S_i\Theta_i\right)\right) + \lambda \left\|\Theta_i\right\|_{\mathrm{od},1} \right] + \beta \sum_{i=2}^{T} \frac{\|\Theta_i - \Theta_{i-1}\|_F^2}{h_i}, \\ \text{s.t.} \quad & \Theta_i \in \mathbb{S}^{p}_{\succ 0}, \quad \forall i \in \{1, 2, ..., T\}. \end{aligned} \qquad [8]$$

Among them, $\beta \geq 0$, and the larger the value of β, the more similar the network structures at adjacent time points. If β tends to infinity, this is equivalent to the static graph estimation in Eq. (7). Additionally, it is important to note that the time of measurement data is not always evenly spaced. Thus, we define $h_i = t_i - t_{i-1}$, representing the time interval between t_i and t_{i-1}. The closer two timestamps are, the more similar their corresponding network structures will be, resulting in larger weights during the optimization process.

In experiments on epithelial-mesenchymal transition (EMT), researchers often apply different inducers to the same type of cells to study their dynamics by observing changes at various scales. While it is possible to model the data independently under different experimental conditions, the inferred network structures often exhibit significant differences.

We observe that the cells at the initial time point are identical. Taking this as a pivot, we can

integrate data from multiple conditions. As shown in Fig.1C, the concentration matrices at the second time point under r different conditions $(\Theta_{21}, \Theta_{22}, \ldots, \Theta_{2r})$ are connected to the concentration matrix at the initial time point Θ_1. Based on this, the optimization problem in Eq. (8) can be further extended as follows:

$$
\begin{aligned}
\min_{\Theta_1,\Theta_{ij}} \quad & -n_1 \left(\log\det\Theta_1 - \operatorname{tr}\left(S_1\Theta_1\right)\right) + \lambda \left\|\Theta_1\right\|_{\mathrm{od},1} + \textstyle\sum_{j=1}^{r}\sum_{i=2}^{T} \beta \frac{\|\Theta_{ij}-\Theta_{i-1,j}\|_F^2}{h_{ij}} \\
& + \textstyle\sum_{j=1}^{r}\sum_{i=2}^{T} \left[-n_{ij}\left(\log\det\Theta_{ij} - \operatorname{tr}\left(S_{ij}\Theta_{ij}\right)\right) + \lambda \left\|\Theta_{ij}\right\|_{\mathrm{od},1} \right], \\
\text{s.t.} \quad & \Theta_1 \in \mathbb{S}_{\succ 0}^{p}, \\
& \Theta_i \in \mathbb{S}_{\succ 0}^{p}, \quad \forall i \in \{2,3,...,T\}, \forall j \in \{1,2,...,r\},
\end{aligned}
\qquad [9]
$$

where $\Theta_1 = \Theta_{11} = \Theta_{12} = \cdots = \Theta_{1r}$, representing the concentration matrix at the initial time point. To solve this optimization problem, we use the Alternating Direction Method of Multipliers (ADMM) algorithm. Firstly, to decompose the optimization problem into a separable form, we introduce auxiliary variables:

$$
\begin{aligned}
Y &= \{Y_1, Y_{21}, Y_{22}, \ldots, Y_{2r}, Y_{31}, Y_{32}, \ldots, Y_{3r}, \ldots, Y_{T1}, Y_{T2}, \ldots, Y_{Tr}\}, \\
Z &= \{Z_{21}, Z_{22}, \ldots, Z_{2r}, Z_{31}, Z_{32}, \ldots, Z_{3r}, \ldots, Z_{T1}, Z_{T2}, \ldots, Z_{Tr}\}.
\end{aligned}
$$

Then, we can write the equivalent problem of the above optimization problem Eq. (9):

$$
\begin{aligned}
\min_{\Theta_1,\Theta_{ij}} \quad & -n_1(\log\det\Theta_1 - \operatorname{tr}(S_1\Theta_1)) + \lambda \parallel Y_1 \parallel_{\mathrm{od},1} \\
& + \textstyle\sum_{j=1}^{r}\sum_{i=2}^{T} \left(-n_{ij}\left(\log\det\Theta_{ij} - \operatorname{tr}\left(S_{ij}\Theta_{ij}\right)\right) + \lambda \parallel Y_{ij} \parallel_{\mathrm{od},1} + \beta \frac{\|Z_{ij}\|_F^2}{h_{ij}} \right), \\
\text{s.t.} \quad & \Theta_1 \in \mathbb{S}_{\succ 0}^{p}, \\
& Y_1 = \Theta_1, \\
& \Theta_i \in \mathbb{S}_{\succ 0}^{p}, \quad \forall i \in \{2,3,...,T\}, \forall j \in \{1,2,...,r\}, \\
& (Y_{i1}, Y_{i2}, ..., Y_{ir}) = (\Theta_{i1}, \Theta_{i2}, ..., \Theta_{ir}), \quad \forall i \in \{2,3,...,T\}, \\
& (Z_{i1}, Z_{i2}, ..., Z_{ir}) = (\Theta_{i1} - \Theta_{i-1,1}, \Theta_{i2} - \Theta_{i-1,2}, ..., \Theta_{i,r} - \Theta_{i-1,r}), \quad \forall i \in \{2,3,...,T\}.
\end{aligned}
\qquad [10]
$$

The corresponding augmented Lagrangian function is expressed as follows:

$$
\begin{aligned}
\mathcal{L}_\rho(\Theta, Y, Z, U, V) &= -n_1(\log\det\Theta_1 - \operatorname{tr}(S_1\Theta_1)) + \lambda \parallel Y_1 \parallel_{\mathrm{od},1} \\
&+ \sum_{j=1}^{r}\sum_{i=2}^{T}\left(-n_{ij}\left(\log\det\Theta_{ij} - \operatorname{tr}\left(S_{ij}\Theta_{ij}\right)\right) + \lambda \parallel Y_{ij} \parallel_{\mathrm{od},1} + \beta\frac{\|Z_{ij}\|_F^2}{h_{ij}}\right) \\
&+ \langle U_1, \Theta_1 - Y_1\rangle + \sum_{j=1}^{r}\sum_{i=2}^{T}\left(\langle U_{ij}, \Theta_{ij} - Y_{ij}\rangle + \langle V_{ij}, \Theta_{ij} - \Theta_{i-1,j} - Z_{ij}\rangle\right) \\
&+ \frac{\rho}{2}\|\Theta_1 - Y_1\|_F^2 + \frac{\rho}{2}\sum_{j=1}^{r}\sum_{i=2}^{T}\left(\|\Theta_{ij} - Y_{ij}\|_F^2 + \|\Theta_{ij} - \Theta_{i-1,j} - Z_{ij}\|_F^2\right).
\end{aligned}
\tag{11}
$$

Here, $\langle\cdot,\cdot\rangle$ denotes the Frobenius inner product between two matrices A and B: $\langle A, B\rangle = \operatorname{tr}(A^T B) = \sum_i\sum_j a_{ij}b_{ij}$. U_{ij} and V_{ij} represent the Lagrange multipliers for Y_{ij} and Z_{ij}, respectively. $\rho > 0$ is a penalty parameter.

To simplify the formulation, we rescale the Lagrange multipliers as $\tilde{U}_{ij} = \frac{U_{ij}}{\rho}$ and $\tilde{V}_{ij} = \frac{V_{ij}}{\rho}$. Substituting these into the Lagrangian yields:

$$
\begin{aligned}
&\sum_{j=1}^{r}\sum_{i=2}^{T}\langle U_{ij}, \Theta_{ij} - Y_{ij}\rangle + \frac{\rho}{2}\sum_{j=1}^{r}\sum_{i=2}^{T}\|\Theta_{ij} - Y_{ij}\|_F^2 \\
&= \rho\sum_{j=1}^{r}\sum_{i=2}^{T}\left\langle \tilde{U}_{ij}, \Theta_{ij} - Y_{ij}\right\rangle + \frac{\rho}{2}\sum_{j=1}^{r}\sum_{i=2}^{T}\|\Theta_{ij} - Y_{ij}\|_F^2 \\
&= \frac{\rho}{2}\sum_{j=1}^{r}\sum_{i=2}^{T}\left(\|\Theta_{ij} - Y_{ij}\|_F^2 + 2\left\langle \tilde{U}_{ij}, \Theta_{ij} - Y_{ij}\right\rangle\right) \\
&= \frac{\rho}{2}\sum_{j=1}^{r}\sum_{i=2}^{T}\left(\|\Theta_{ij} - Y_{ij}\|_F^2 + 2\left\langle \tilde{U}_{ij}, \Theta_{ij} - Y_{ij}\right\rangle + \parallel \tilde{U}_{ij} \parallel_F^2 - \parallel \tilde{U}_{ij} \parallel_F^2\right) \\
&= \frac{\rho}{2}\sum_{j=1}^{r}\sum_{i=2}^{T}\left(\parallel \Theta_{ij} - Y_{ij} + \tilde{U}_{ij} \parallel_F^2 - \parallel \tilde{U}_{ij} \parallel_F^2\right).
\end{aligned}
\tag{12}
$$

Similarly, we have:

$$
\begin{aligned}
&\sum_{j=1}^{r}\sum_{i=2}^{T}\langle V_{ij}, \Theta_{ij} - \Theta_{i-1,j} - Z_{ij}\rangle + \frac{\rho}{2}\sum_{j=1}^{r}\sum_{i=2}^{T}\|\Theta_{ij} - \Theta_{i-1,j} - Z_{ij}\|_F^2 \\
&= \frac{\rho}{2}\sum_{j=1}^{r}\sum_{i=2}^{T}\left(\parallel \Theta_{ij} - \Theta_{i-1,j} - Z_{ij} + \tilde{V}_{ij} \parallel_F^2 - \parallel \tilde{V}_{ij} \parallel_F^2\right).
\end{aligned}
\tag{13}
$$

Substituting Eq. (12) and Eq. (13) into Eq. (11), we obtain:

$$\begin{aligned}
&\mathcal{L}_\rho(\Theta, Y, Z, U, V) \\
&= -n_1(\log\det\Theta_1 - \operatorname{tr}(S_1\Theta_1)) + \lambda \parallel Y_1 \parallel_{\text{od},1} \\
&+ \textstyle\sum_{j=1}^{r}\sum_{i=2}^{T}\left(-n_{ij}\left(\log\det\Theta_{ij} - \operatorname{tr}\left(S_{ij}\Theta_{ij}\right)\right) + \lambda \parallel Y_{ij} \parallel_{\text{od},1} + \beta\frac{\|Z_{ij}\|_F^2}{h_{ij}}\right). \\
&+ \tfrac{\rho}{2}\left(\parallel \Theta_1 - Y_1 + \tilde{U}_1 \parallel_F^2 - \parallel \tilde{U}_1 \parallel_F^2\right) \\
&+ \tfrac{\rho}{2}\textstyle\sum_{j=1}^{r}\sum_{i=2}^{T}\left(\parallel \Theta_{ij} - Y_{ij} + \tilde{U}_{ij} \parallel_F^2 - \parallel \tilde{U}_{ij} \parallel_F^2\right) \\
&+ \tfrac{\rho}{2}\textstyle\sum_{j=1}^{r}\sum_{i=2}^{T}\left(\parallel \Theta_{ij} - \Theta_{i-1,j} - Z_{ij} + \tilde{V}_{ij} \parallel_F^2 - \parallel \tilde{V}_{ij} \parallel_F^2\right).
\end{aligned} \qquad [14]$$

The ADMM algorithm minimizes $\mathcal{L}_\rho(\Theta, Y, Z, \tilde{U}, \tilde{V})$ by alternately updating the variables $\Theta, Y, Z, \tilde{U}$, and $\tilde{V}$. Below, we derive the update equations for these parameters step by step:

1. Updating Θ: For each case j ($j \in \{1, 2, ..., r\}$) and each time point i ($i \in \{2, 3, ..., T-1\}$), the optimization problem for Θ_{ij} is formulated as follows, fixing all other variables:

$$\begin{aligned}
\Theta_{ij}^{k+1} = \underset{\Theta_{ij}}{argmin} \quad & -n_{ij}\left(\log\det\Theta_{ij} - \operatorname{tr}\left(S_{ij}\Theta_{ij}\right)\right) + \tfrac{\rho}{2} \parallel \Theta_{ij} - (Y_{ij}^k - \tilde{U}_{ij}^k) \parallel_F^2 \\
& + \tfrac{\rho}{2} \parallel \Theta_{ij} - (\Theta_{i-1,j}^{k+1} + Z_{ij}^k - \tilde{V}_{ij}^k) \parallel_F^2 \\
& + \tfrac{\rho}{2} \parallel \Theta_{ij} - (\Theta_{i+1,j}^{k} - Z_{i+1,j}^k + \tilde{V}_{i+1,j}^k) \parallel_F^2 .
\end{aligned}$$

This is equivalent to:

$$\begin{aligned}
\Theta_{ij}^{k+1} = \underset{\Theta_{ij}}{argmin} \quad & -\log\det\Theta_{ij} + \operatorname{tr}\left(S_{ij}\Theta_{ij}\right) + \tfrac{\rho}{2n_{ij}} \parallel \Theta_{ij} - (Y_{ij}^k - \tilde{U}_{ij}^k) \parallel_F^2 \\
& + \tfrac{\rho}{2n_{ij}} \parallel \Theta_{ij} - (\Theta_{i-1,j}^{k+1} + Z_{ij}^k - \tilde{V}_{ij}^k) \parallel_F^2 \\
& + \tfrac{\rho}{2n_{ij}} \parallel \Theta_{ij} - (\Theta_{i+1,j}^{k} - Z_{i+1,j}^k + \tilde{V}_{i+1,j}^k) \parallel_F^2 \\
& - \tfrac{\rho}{6n_{ij}} \parallel (Y_{ij}^k - \tilde{U}_{ij}^k) - (\Theta_{i-1,j}^{k+1} + Z_{ij}^k - \tilde{V}_{ij}^k) \parallel_F^2 \\
& - \tfrac{\rho}{6n_{ij}} \parallel (Y_{ij}^k - \tilde{U}_{ij}^k) - (\Theta_{i+1,j}^{k} - Z_{i+1,j}^k + \tilde{V}_{i+1,j}^k) \parallel_F^2 \\
& - \tfrac{\rho}{6n_{ij}} \parallel (\Theta_{i-1,j}^{k+1} + Z_{ij}^k - \tilde{V}_{ij}^k) - (\Theta_{i+1,j}^{k} - Z_{i+1,j}^k + \tilde{V}_{i+1,j}^k) \parallel_F^2 .
\end{aligned}$$

After simplification, we derive the following formula:

$$\Theta_{ij}^{k+1} = \underset{\Theta_{ij}}{argmin} \quad -\log\det\Theta_{ij} + \operatorname{tr}\left(S_{ij}\Theta_{ij}\right) + \tfrac{3\rho}{2n_{ij}} \parallel \Theta_{ij} - A \parallel_F^2,$$

where

$$A = \frac{(Y_{ij}^k - \tilde{U}_{ij}^k) + (\Theta_{i-1,j}^{k+1} + Z_{ij}^k - \tilde{V}_{ij}^k) + (\Theta_{i+1,j}^k - Z_{i+1,j}^k + \tilde{V}_{i+1,j}^k)}{3}.$$

Given the symmetry of Θ_{ij}, this is further equivalent to:

$$\Theta_{ij}^{k+1} = \underset{\Theta_{ij}}{argmin} \quad -\log\det\Theta_{ij} + \operatorname{tr}(S_{ij}\Theta_{ij}) + \eta \parallel \Theta_{ij} - \tfrac{A+A^T}{2} \parallel_F^2, \qquad [15]$$

where $\eta = \frac{3\rho}{2n_{ij}}$. Taking the derivative with respect to Θ_{ij} yields:

$$\Theta_{ij}^{-1} - 2\eta\Theta_{ij} = S_{ij} - \eta(A + A^T). \qquad [16]$$

Based on the commutative property of matrices, it can be deduced that Θ_{ij} and $S_{ij} - \eta(A + A^T)$ share the same eigenvectors. pecifically, according to Equation Eq. (16), we have:

$$\Theta_{ij}\left[S_{ij} - \eta(A + A^T)\right] = \left[S_{ij} - \eta(A + A^T)\right]\Theta_{ij}.$$

. By performing eigendecomposition on $S_{ij} - \eta(A + A^T)$, we obtain:

$$S_{ij} - \eta(A + A^T) = Q\Lambda Q^T,$$

where Q is an orthogonal matrix whose columns are the eigenvectors of $S_{ij} - \eta(A + A^T)$, and Λ is a diagonal matrix with its diagonal elements corresponding to the eigenvalues, i.e., $\Lambda_{ii} = \lambda_i$. Using the orthogonal matrix Q, we can similarly diagonalize Θ_{ij}:

$$\Theta_{ij} = Q\tilde{\Lambda}Q^T,$$

where $\tilde{\Lambda}$ is a diagonal matrix with diagonal elements $\tilde{\Lambda}_{ii} = \tilde{\lambda}_i$. Substituting into Equation Eq. (16), we rewrite it as:

$$(Q\tilde{\Lambda}Q^T)^{-1} - 2\eta Q\tilde{\Lambda}Q^T = Q\Lambda Q^T.$$

Simplifying this expression yields:

$$Q\tilde{\Lambda}^{-1}Q^T - 2\eta Q\tilde{\Lambda}Q^T = Q\Lambda Q^T.$$

For each pair of eigenvalues λ_i and $\tilde{\lambda}_i$, the following relationship holds:

$$\frac{1}{\tilde{\lambda}_i} - 2\eta\tilde{\lambda}_i = \lambda_i. \quad [17]$$

Solving Equation Eq. (17), we obtain:

$$\tilde{\lambda}_i = \frac{-\lambda_i + \sqrt{\lambda_i^2 + 8\eta}}{4\eta}.$$

Consequently, the update formula for Θ_{ij} is derived as:

$$\Theta_{ij}^{k+1} = \frac{1}{4\eta} Q(-\Lambda + \sqrt{\Lambda^2 + 8\eta I})Q^T. \quad [18]$$

For $i = T$, the optimization problem for Θ_{Tj} can be expressed as:

$$\begin{aligned}\Theta_{Tj}^{k+1} = \underset{\Theta_{Tj}}{argmin} \quad & -\log\det\Theta_{Tj} + \operatorname{tr}(S_{Tj}\Theta_{Tj}) + \tfrac{\rho}{2n_{Tj}} \parallel \Theta_{Tj} - (Y_{Tj}^k - \tilde{U}_{Tj}^k) \parallel_F^2 \\ & + \tfrac{\rho}{2n_{Tj}} \parallel \Theta_{Tj} - (\Theta_{T-1,j}^{k+1} + Z_{Tj}^k - \tilde{V}_{Tj}^k) \parallel_F^2 \\ & - \tfrac{\rho}{4n_T} \parallel (Y_{Tj}^k - \tilde{U}_{Tj}^k) - (\Theta_{T-1,j}^{k+1} + Z_{Tj}^k - \tilde{V}_{Tj}^k) \parallel_F^2,\end{aligned}$$

this is equivalent to:

$$\Theta_{Tj}^{k+1} = \underset{\Theta_{Tj}}{argmin} \quad -\log\det\Theta_{Tj} + \operatorname{tr}(S_{Tj}\Theta_{Tj}) + \tfrac{\rho}{n_{Tj}} \parallel \Theta_{Tj} - A \parallel_F^2,$$

where

$$A = \frac{(Y_{Tj}^k - \tilde{U}_{Tj}^k) + (\Theta_{T-1,j}^{k+1} + Z_{Tj}^k - \tilde{V}_{Tj}^k)}{2}.$$

Considering the symmetry of Θ_{Tj}, this can be further simplified as:

$$\Theta_{Tj}^{k+1} = \underset{\Theta_{Tj}}{argmin} \quad -\log\det\Theta_{Tj} + \mathrm{tr}\left(S_{Tj}\Theta_{Tj}\right) + \eta_{Tj} \parallel \Theta_{Tj} - \tfrac{A+A^T}{2} \parallel_F^2, \tag{19}$$

where $\eta_{Tj} = \frac{\rho}{n_{Tj}}$. Taking the derivative of Equation Eq. (19) with respect to Θ_{Tj}, we have:

$$\Theta_{Tj}^{-1} - 2\eta_{Tj}\Theta_{Tj} = S_{Tj} - \eta_{Tj}(A + A^T).$$

From this, the update formula for Θ_{Tj} can be derived as:

$$\Theta_{Tj}^{k+1} = \frac{1}{4\eta_{Tj}} Q(-\Lambda + \sqrt{\Lambda^2 + 8\eta_{Tj} I})Q^T, \tag{20}$$

where Q and Λ satisfy the eigendecomposition

$$S_{Tj} - \eta_{Tj}(A + A^T) = Q\Lambda Q^T,$$

and $A = \frac{(Y_{Tj}^k - \tilde{U}_{Tj}^k) + (\Theta_{T-1,j}^{k+1} + Z_{Tj}^k - \tilde{V}_{Tj}^k)}{2}$.

For $i = 1$, the optimization problem can be formulated as:

$$\begin{aligned} \Theta_1^{k+1} = \underset{\Theta_1}{argmin} \quad & -\log\det\Theta_1 + \mathrm{tr}\left(S_1\Theta_1\right) + \tfrac{\rho}{2n_1} \parallel \Theta_1 - (Y_1^k - \tilde{U}_1^k) \parallel_F^2 \\ & + \tfrac{\rho}{2n_1} \parallel \Theta_1 - (\Theta_{21}^k - Z_{21}^k + \tilde{V}_{21}^k) \parallel_F^2 \\ & + \tfrac{\rho}{2n_1} \parallel \Theta_1 - (\Theta_{22}^k - Z_{22}^k + \tilde{V}_{22}^k) \parallel_F^2 \\ & + \tfrac{\rho}{2n_1} \parallel \Theta_1 - (\Theta_{23}^k - Z_{23}^k + \tilde{V}_{23}^k) \parallel_F^2 . \end{aligned} \tag{21}$$

Define

$$A = \frac{(Y_1^k - \tilde{U}_1^k) + (\Theta_{21}^k - Z_{21}^k + \tilde{V}_{21}^k) + (\Theta_{22}^k - Z_{22}^k + \tilde{V}_{22}^k) + (\Theta_{23}^k - Z_{23}^k + \tilde{V}_{23}^k)}{4}.$$

The optimization problem in Eq. (21) is equivalent to:

$$\Theta_1^{k+1} = \underset{\Theta_1}{argmin} \quad -\log\det\Theta_1 + \mathrm{Tr}\,(S_1\Theta_1) + \eta_1 \parallel \Theta_1 - \tfrac{A+A^T}{2} \parallel_F^2,$$

where $\eta_1 = \frac{4\rho}{2n_1}$. Thus, the update formula for Θ_1 is:

$$\Theta_1^{k+1} = \tfrac{1}{4\eta_1} Q(-\Lambda + \sqrt{\Lambda^2 + 8\eta_1 I})Q^T, \qquad [22]$$

where $Q\Lambda Q^T$ is the eigendecomposition of $S_1 - \eta_1(A + A^T)$.

2. Updating Y: For $j = 1, 2, ..., r$ and $i = 2, ..., T$, fixing other variables, the optimization problem with respect to Y_{ij} can be expressed as:

$$Y_{ij}^{k+1} = \underset{Y_{ij}}{argmin} \ \ \tfrac{\rho}{2} \parallel \Theta_{ij}^{k+1} - Y_{ij} + \tilde{U}_{ij}^k \parallel_F^2 + \lambda \parallel Y_{ij} \parallel_{od,1} .$$

This problem can be solved using the soft-thresholding operator. Specifically, for each element y_{mn} of Y_{ij}, the corresponding element $\theta_{mn} + \tilde{u}_{mn}$ in $\Theta_{ij}^{k+1} + \tilde{U}_{ij}^k$ satisfies the following derivative conditions with respect to y_{mn}:

$$\begin{aligned} \rho(y_{mn} - (\theta_{mn} + \tilde{u}_{mn})) + \lambda &= 0, \quad \text{if } y_{mn} > 0, \\ \rho(y_{mn} - (\theta_{mn} + \tilde{u}_{mn})) - \lambda &= 0, \quad \text{if } y_{mn} < 0. \end{aligned}$$

Simplifying these equations gives:

$$\begin{aligned} y_{mn} &= \theta_{mn} + \tilde{u}_{mn} - \tfrac{\lambda}{\rho}, \quad \text{if } \ y_{mn} > 0, \\ y_{mn} &= \theta_{mn} + \tilde{u}_{mn} + \tfrac{\lambda}{\rho}, \quad \text{if } \ y_{mn} < 0. \end{aligned}$$

For y_{mn} to remain positive, the condition $\theta_{mn} + \tilde{u}_{mn} - \frac{\lambda}{\rho} > 0$ must hold; otherwise, $y_{mn} = 0$. Similarly, for y_{mn} to remain negative, the condition $\theta_{mn} + \tilde{u}_{mn} + \frac{\lambda}{\rho} < 0$ must hold; otherwise, $y_{mn} = 0$. Thus, y_{mn} can be expressed as:

$$y_{mn} = sign(\theta_{mn} + \tilde{u}_{mn}) * max(|\theta_{mn} + \tilde{u}_{mn}| - \frac{\lambda}{\rho}, 0).$$

For Y_{ij}, the solution is:

$$Y_{ij}^{k+1} = sign(\Theta_{ij}^{k+1} + U_{ij}^{k}) * max(|\Theta_{ij}^{k+1} + U_{ij}^{k}| - \tfrac{\lambda}{\rho}, 0). \quad [23]$$

When $i = 1$, the solution for Y_1 is similar and can be written as:

$$Y_{1}^{k+1} = sign(\Theta_{1}^{k+1} + U_{1}^{k}) * max(|\Theta_{1}^{k+1} + U_{1}^{k}| - \tfrac{\lambda}{\rho}, 0). \quad [24]$$

3. Updating Z: For $j = 1, 2, ..., r$ and $i = 2, ..., T$, with other variables fixed, the optimization problem for Z_{ij} is given by:

$$Z_{ij}^{k+1} = \underset{Z_{ij}}{argmin} \;\; \beta \tfrac{\|Z_{ij}\|_F^2}{h_{ij}} + \tfrac{\rho}{2} \parallel \Theta_{ij}^{k+1} - \Theta_{i-1,j}^{k+1} - Z_{ij} + \tilde{V}_{ij}^{k} \parallel_F^2 .$$

Taking the derivative with respect to Z_{ij}, we obtain:

$$\tfrac{2\beta}{h_{ij}} Z_{ij} + \rho(Z_{ij} - \Theta_{ij}^{k+1} + \Theta_{i-1,j}^{k+1} - \tilde{V}_{ij}^{k}) = 0,$$

Simplifying this, we get the iteration formula for Z_{ij}:

$$Z_{ij}^{k+1} = \tfrac{\rho h_{ij}}{\rho h_{ij} + 2\beta}(\Theta_{ij}^{k+1} - \Theta_{i-1,j}^{k+1} + \tilde{V}_{ij}^{k}). \quad [25]$$

4. Updating $\tilde{U}$ and $\tilde{V}$: Taking the derivatives of $\mathcal{L}_\rho(\Theta, Y, Z, \tilde{U}, \tilde{V})$ in Eq. (14) with respect to $\tilde{V}_{ij}$ and $\tilde{U}_{ij}$, we obtain:

$$\begin{aligned}
\tfrac{\partial \mathcal{L}_\rho}{\partial \tilde{U}_1} &= \rho(\Theta_1^{k+1} - Y_1^{k+1}), \\
\tfrac{\partial \mathcal{L}_\rho}{\partial \tilde{U}_{ij}} &= \rho(\Theta_{ij}^{k+1} - Y_{ij}^{k+1}), \\
\tfrac{\partial \mathcal{L}_\rho}{\partial \tilde{V}_{ij}} &= \rho(\Theta_{ij}^{k+1} - \Theta_{i-1,j}^{k+1} - Z_{ij}^{k+1}),
\end{aligned}$$

Thus, the iteration formulas for $\tilde{V}_{ij}$ and $\tilde{U}_{ij}$ are:

$$\begin{aligned}
\tilde{U}_1^{k+1} &= \tilde{U}_1^k + \rho(\Theta_1^{k+1} - Y_1^{k+1}),\\
\tilde{U}_{ij}^{k+1} &= \tilde{U}_{ij}^k + \rho(\Theta_{ij}^{k+1} - Y_{ij}^{k+1}), \quad \forall i \in \{2,3,...,T\}, \forall j \in \{1,2,...,r\},\\
\tilde{V}_{ij}^{k+1} &= \tilde{V}_{ij}^k + \rho(\Theta_{ij}^{k+1} - \Theta_{i-1,j}^{k+1} - Z_{ij}^{k+1}), \quad \forall i \in \{2,3,...,T\}, \forall j \in \{1,2,...,r\}.
\end{aligned} \tag{26}$$

4. Estimation of the diffusion coefficient d

Given the N-dimensional gene expression data $\{\boldsymbol{x}_{t_1}, \boldsymbol{x}_{t_2}, ..., \boldsymbol{x}_{t_l}\}$ with length l, we assume that the data are generated by a stochastic dynamical system and estimate the diffusion coefficient d from it by the mean squared displacement (5). External and internal noise can both be critical to system dynamics, and we first consider the external noise form of Langevin equation,

$$\frac{\mathrm{d}\boldsymbol{x}}{\mathrm{d}t} = \boldsymbol{F}(\boldsymbol{x}) + \frac{1}{\gamma}\boldsymbol{\xi}(t). \tag{27}$$

Here, $\boldsymbol{F}(\boldsymbol{x})$ represents the driving force of the system, $\boldsymbol{\xi}(t)$ denotes Gaussian white noise with zero mean, obeying $< \boldsymbol{\xi}(t) >= 0$, $< \boldsymbol{\xi}(t), \boldsymbol{\xi}(s) >= 2\gamma k_B T\delta(t-s)$, where γ is the effective friction, k_B is Boltzmann's constant and T is the temperature. Then from $d = \frac{k_B T}{\gamma}$, it can be written as:

$$\frac{\mathrm{d}\boldsymbol{x}}{\mathrm{d}t} = \boldsymbol{F}(\boldsymbol{x}) + \sqrt{2d}\boldsymbol{\eta}(t). \tag{28}$$

Then we can obtain the iterative formula for Eq. (28),

$$\boldsymbol{x}_{t_{i+1}} = \boldsymbol{x}_{t_i} + \boldsymbol{F}\left(\boldsymbol{x}_{t_i}\right)\Delta t + \sqrt{2d\Delta t}\boldsymbol{Z}, \quad \forall i \in \{1, \cdots, l-1\}, \tag{29}$$

where $\boldsymbol{Z}$ is a standard normal random vector, implying $\frac{x_{t_{i+1}}^{(k)} - x_{t_i}^{(k)}}{\sqrt{2d\Delta t}} - \sqrt{\frac{\Delta t}{2d}} F^{(k)}\left(\boldsymbol{x}_{t_i}\right)$ is a standard normal random variable for all $i \in \{1, 2, ..., l-1\}$, $x_{t_i}^{(k)}$ is the kth component of $\boldsymbol{x}_{t_i}$. Assuming the time step Δt is infinitesimal, $\sqrt{\frac{\Delta t}{2d}} F^{(k)}\left(\boldsymbol{x}_{t_i}\right)$ can be ignored, and the remaining $\frac{x_{t_{i+1}}^{(k)} - x_{t_i}^{(k)}}{\sqrt{2d\Delta t}}$ can be considered as a standard normal random variable for all $i \in \{1, 2, ..., l-1\}$. Using the sample

variance to approximate the true variance, we can obtain

$$\frac{1}{2\hat{d}_k \Delta t(l-1)} \sum_{i=1}^{l-1} \left(x_{t_{i+1}}^{(k)} - x_{t_i}^{(k)} \right)^2 = 1, \tag{30}$$

$$\hat{d}_k = \frac{1}{2\Delta t(l-1)} \sum_{i=1}^{l-1} \left(x_{t_{i+1}}^{(k)} - x_{t_i}^{(k)} \right)^2, \tag{31}$$

where $\hat{d}_k$ is the estimation of d. We can get N estimations of d ($\hat{d}_k$, $k \in \{1, 2, ..., N\}$). Thus the final estimation of d can be obtained from the average of $\hat{d}_k$,

$$\hat{d} = \frac{1}{N} \sum_{k=1}^{N} \hat{d}_k. \tag{32}$$

For the internal noise form of the Langevin equation,

$$\frac{\mathrm{d}\boldsymbol{x}}{\mathrm{d}t} = \boldsymbol{F}(\boldsymbol{x}) + \boldsymbol{\xi}(\boldsymbol{x}, t), \tag{33}$$

where the noise is usually assumed to be N-dimensional independent Gaussian white noise, depending on the variables $\boldsymbol{x}$,

$$< \boldsymbol{\xi}(\boldsymbol{x}, t) > = 0, \tag{34}$$

$$< \boldsymbol{\xi}(\boldsymbol{x}, t), \boldsymbol{\xi}(\boldsymbol{x}, s) > = 2\boldsymbol{d}(\boldsymbol{x})\delta(t-s). \tag{35}$$

Therefore, $\boldsymbol{d}(\boldsymbol{x})$ has the following form,

$$\boldsymbol{d}(\boldsymbol{x}) = \begin{pmatrix} d_1(\boldsymbol{x}) & 0 & \cdots & 0 \\ 0 & d_2(\boldsymbol{x}) & \cdots & 0 \\ \vdots & \vdots & \ddots & \vdots \\ 0 & 0 & \cdots & d_N(\boldsymbol{x}) \end{pmatrix}. \tag{36}$$

Then, the iterative formula of Eq. (33) can be written as,

$$x_{t+1}^{(k)} = x_t^{(k)} + F^{(k)}(\boldsymbol{x}_t)\Delta t + \sqrt{2d_k(\boldsymbol{x})\Delta t} Z^{(k)}, \quad \forall k \in \{1, 2, ..., d\}, \tag{37}$$

where $Z^{(k)}$ is a standard normal random variable. For the extrinsic noise form of the Langevin equation, the friction γ is inversely proportional to the diffusion coefficient d, so we assume that this property holds for the intrinsic noise case to facilitate the estimation of γ, i.e.,

$$\frac{1}{\gamma} \propto \frac{1}{N} \sum_{k=1}^{N} d_k(\boldsymbol{x}), \tag{38}$$

and we can also use the same method to estimate kth diffusion term,

$$\hat{d}_k = \frac{1}{2\Delta t(l-1)} \sum_{i=1}^{l-1} \left(x_{t_{i+1}}^{(k)} - x_{t_i}^{(k)} \right)^2. \tag{39}$$

For convenience, we take the scale factor as 1, i.e.,

$$\hat{\gamma} = \frac{1}{\frac{1}{N}\sum_{k=1}^{N} \hat{d}_k}. \tag{40}$$

In this way, we are able to estimate the diffusion coefficient d of the system either from external or intrinsic noise.

5. Energy landscape construction

To investigate the stochastic dynamics of the system, we add an external noise term into Eq. 1 in the main text:

$$\tfrac{d\boldsymbol{x}(t)}{dt} = \boldsymbol{F}(\boldsymbol{x}(t)) + \boldsymbol{\Gamma}(t), \tag{41}$$

where $\boldsymbol{x}(t) = (x_1(t), x_2(t), ..., x_N(t))^T$, $\boldsymbol{F}(\boldsymbol{x}(t))$ is a vector composed of $F_i(\boldsymbol{x}(t))$, $i = 1, 2, ..., N$, representing the driving force of the system. $\boldsymbol{\Gamma}(t) = (\Gamma_1(t), \Gamma_2(t), ..., \Gamma_N(t))^T$ is N-dimensional independent Gaussian white noise, satisfying $\mathbb{E}[\Gamma_i(t)] = 0$ and $\mathbb{E}[\Gamma_i(t)\Gamma_j(t')] = 2d\delta_{ij}\delta_0(t-t')$ for arbitrary t and t'. Here, d is the constant diffusion coefficien, which can be estimated from the data.

Only when $i = j$, $\delta_{ij} = 1$, otherwise $\delta_{ij} = 0$, indicating that the noises are independent for different i and j. $\delta_0(t - t')$ is Dirac Delta function, which implies that for one variable, the noises at different times are independent.

The time evolution of this dynamical system is determined by the Fokker-Planck equation or probabilistic diffusion equation:

$$\frac{\partial p(\boldsymbol{x},t)}{\partial t} = -\sum_i \frac{\partial}{\partial x_i}[F_i(\boldsymbol{x},t)p(\boldsymbol{x},t)] + d\sum_i \sum_j \frac{\partial^2}{\partial x_i \partial x_j} p(\boldsymbol{x},t), \qquad [42]$$

where $p(\boldsymbol{x}, t)$ is the probability density function of system state at time t. And this partial differential equation is difficult to solve due to its high dimensionality and nonlinearity. Here, the Gaussian distribution along the deterministic trajectory is employed to approximate the time evolution of the density function of the system. Concerning a Gaussian distribution, once two moments, namely the mean and the variance, are determined, the probability distribution can be obtained. Hence, we only have to calculate the mean $\bar{\boldsymbol{x}}(t)$ and the variance $\boldsymbol{\Sigma}(t)$. When the diffusion coefficient d is small, the moment equations satisfy the subsequent equations (6, 7):

$$\dot{\bar{\boldsymbol{x}}}(t) = \boldsymbol{F}[\bar{\boldsymbol{x}}(t)], \qquad [43]$$

$$\dot{\boldsymbol{\Sigma}}(t) = \boldsymbol{\Sigma}(t)\boldsymbol{A}^T(t) + \boldsymbol{A}(t)\boldsymbol{\Sigma}(t) + 2d \cdot \boldsymbol{I}. \qquad [44]$$

Here, $\boldsymbol{I}$ is identity matrix, and $\boldsymbol{A}(t)$ is the jacobian matrix of $\boldsymbol{F}[\bar{\boldsymbol{x}}(t)]$ when $\boldsymbol{x}(t)$ is equal to the solution of the deterministic equation $\bar{\boldsymbol{x}}(t)$, i.e., $A_{ij}(t) = \frac{\partial F_i(\boldsymbol{x}(t))}{\partial x_j}|_{\boldsymbol{x}(t)=\bar{\boldsymbol{x}}(t)}$. In this work, as the driving force $\boldsymbol{F}$ in Eq. (43) is modeled by Nerual ODE, we uniformly and randomly pick points as initial values for its solution. When the number of iterations is ample enough, the output of Neural ODE gradually stabilizes, thereby obtaining the stable state. And the Jacobi matrix $\boldsymbol{A}(t)$ in Eq. (44) is approximated by the secant slope. Then, the probability density distribution of the system at the steady state can be expressed as:

$$p_{ss}(\boldsymbol{x}) = \frac{1}{(2\pi)^{N/2}|\boldsymbol{\Sigma}|^{1/2}} \exp\{-\frac{1}{2}(\boldsymbol{x} - \mu)^T \boldsymbol{\Sigma}^{-1}(\boldsymbol{x} - \mu)\}, \qquad [45]$$

where μ and Σ are the solutions of Eq. (43) and Eq. (44) when $t \to \infty$, respectively. If Eq. (43) has multiple stable states, the final probability density function of the system is depicted by the weighted sum of Gaussian distributions (2, 8, 9):

$$p_{ss}(\boldsymbol{x}) = \sum_{j=1}^{M} \phi^j p_{ss}^j(\boldsymbol{x}). \quad [46]$$

Among which, M is the number of the stable states. $p_{ss}^j(\boldsymbol{x})$ represents the density function of the j-th stable state, and ϕ^j denotes the corresponding weight. The weight is estimated through the statistics of the frequency of each stable state under a large number of initial conditions.

Finally, we can construct the potential landscape by $U(\boldsymbol{x}) = -\ln p_{ss}(\boldsymbol{x})$ (2, 8, 9). However, for a high-dimensional system, its potential energy landscape is hard to visualize and interpret. We can project the landscape into a low-dimensional space, and the projection matrix is denoted as $\boldsymbol{V}$. Following previous work (8), $\boldsymbol{V}$ can be constituted by the eigenvectors corresponding to the first C largest eigenvalues of the covariance matrix $\boldsymbol{\Sigma}$. For $\forall j \in \{1, 2, ..., M\}$, let $\boldsymbol{Z}^j = \boldsymbol{V}^T \boldsymbol{X}^j$. Then, the mean and covariance matrix of j-th stable state after projection are $\boldsymbol{\mu}_z^j = \boldsymbol{V}^T \boldsymbol{\mu}^j$ and $\boldsymbol{\Sigma}_z^j = \boldsymbol{V}^T \boldsymbol{\Sigma}^j \boldsymbol{V}$, respectively. Correspondingly, the multi-dimensional normal distribution of j-th stable state can be expressed as:

$$p_z^j(\boldsymbol{z}) = \frac{1}{(2\pi)^{C/2} |\boldsymbol{\Sigma}_z^j|^{1/2}} \exp\{-\frac{1}{2}(\boldsymbol{z} - \boldsymbol{\mu}_z^j)^T (\boldsymbol{\Sigma}_z^j)^{-1} (\boldsymbol{x} - \boldsymbol{\mu}_z^j)\}. \quad [47]$$

Hence, the final probability density function after dimension reduction is $p_z(\boldsymbol{z}) = \sum_{j=1}^{M} \phi^j p_z^j(\boldsymbol{z})$, and the potential landscape is $U_z(\boldsymbol{z}) = -\ln p_z(\boldsymbol{z})$.

6. Calculation of $Score_{E \to M}$

The rationale behind the construction of the $Score_{E \to M}$ metric is as follows. In the state space, each point represents a gene expression profile.

In our previous work (10, 11), we observed that reducing the expression of E marker genes typically drives the system toward the M state, whereas reducing the expression of M marker genes tends to shift it in the opposite direction. Building on this empirical regularity,we reasoned that perturbing a specific gene X_i (e.g., knockout) may alter the stability of the E state in different directions along

the E-M axis. If the perturbed E state (after X_i is knocked out) shifts toward the M state, this suggests that X_i plays a critical role in maintaining the E state, i.e., it acts as an E-state gene. Conversely, if the perturbed E state shifts away from the M state, the gene is more likely to be an M-state gene, contributing to mesenchymal characteristics.

To quantify this, we project the new steady state obtained after single-gene knockout onto the line connecting the E and M states. The sign of the projection indicates whether the perturbation (knock out) moves the system toward M (negative score) or away from M (positive score). This design allows us to distinguish M-state–critical genes from E-state–critical genes in a systematic manner.

Specifically, we conduct single-gene knockout experiments in the M state and the E state, respectively. for instance, by knocking out the gene x_j, the newly obtained stable states are denoted as $\boldsymbol{x}_{ss,j}^{M}$ and $\boldsymbol{x}_{ss,j}^{E}$, respectively. Below, we introduce the indicator $Score_{E\to M}$ to quantitatively characterize the role of the gene in the transition from the E state to the M state. Specifically, for the knockout experiment in the M state, we project the new stable state $\boldsymbol{x}_{ss,j}^{M}$ onto the direction jointly determined by $\boldsymbol{x}_{ss}^{M}$ and $\boldsymbol{x}_{ss}^{E}$ in the PCA dimensionality reduction space. The distance between the projection point and the M state is the absolute value of $Score_{E\to M}(x_j)$, namely $|Score_{E\to M}(x_j)|$. Based on the positions of the projection point, the M state and the E state, we can determine the sign of $Score_{E\to M}(x_j)$: If the projection point is closer to the E state than the M state, the sign is positive; otherwise, it is negative. Similarly, for the knockout experiment conducted in the E state, we define $|Score_{E\to M}(x_j)|$ as the distance between the projection point and the E state. Moreover, if the projection point is farther from the M state compared to the E state, $Score_{E\to M}(x_j)$ takes a positive value. Essentially, the indicator $Score_{E\to M}(x_j)$ measures the extent to which the system moves away from the M state and evolves in a more epithelial-like direction after the knockout of the gene x_j. Therefore, $Score_{E\to M}(x_j) > 0$ indicates that the gene x_j may play an important role in the transition from the E state to the M state, and the greater the value, the more significant this role becomes.

7. Network Inference on a Feedback-Rich Regulatory Network

To see whether our method works in the complex models with more feedback loops, we applied our GGANO to a network from (10). This network includes 12 transcription factors, 4 mRNAs, and 53 regulatory interactions (Fig.S2A). Among them, there are 10 mesenchymal markers (GSC, FOXC2, VIM, TWIST1, TCF3, TGF-β, ZEB1, ZEB2, SNAI, SNAI2) and 6 epithelial markers (miR-34a, miR-141, miR-145, miR-200, Ovol2, CDH1). Baesd on the Eq. 1 in the main text, the parameters in this model are set as: $a = 0.5$, $b = 0.5$, $S = 0.2$, $n = 4.0$, $k = 1.0$. Compared to the MESC model, this network exhibits more complexity due to the inclusion of many feedback loops. Nevertheless, even at a high noise level ($d = 0.5$), GGM can still accurately infer the undirected structure of the network, as shown in Fig.S2B. Among the 46 inferred regulatory interactions, 41 match the standard structure (gray solid lines), and the remaining 5 interactions are additional predictions (red solid lines). It is worth noting that only one regulatory interaction in the standard structure is not predicted (red dotted line). From the ROC curve in Fig.S2C, it can be witnessed that subsequent to integrating the prior information into the Neural ODE model, the AUC value ascends from 0.83 to 0.96, suggesting that GGANO can notably enhance the accuracy of network inference. As shown in Fig.S2D, the structure inferred by GGANO closely resembles the standard structure, whereas the Neural ODE result (Fig. S2E) deviates more significantly.

8. Application to the dateset of cell fate decisions

We examine a dataset of 48 gene expression profiles throughout mouse pre-implantation development, encompassing developmental stages from the 1-cell to the 64-cell stage (12). During this period, cells undergo two major fate decisions: at the 16-cell stage, cells differentiate into two distinct lineages, the trophectoderm (TE) and the inner cell mass (ICM); and at the 32-cell stage, ICM cells further differentiate into the primitive endoderm (PE) and the epiblast (EPI).

Our method involves several hyperparameters, including λ, β, and $thre$. Specifically, λ controls the sparsity of the inferred network, with larger values leading to sparser structures; β regulates temporal homogeneity, encouraging greater similarity between adjacent networks as its value increases; and

thre is a threshold used to distinguish zero from non-zero elements in the precision matrix Θ. To assess their effects on network inference, we systematically varied these hyperparameters. As shown in Fig. 5D (upper panel), the precision matrices Θ of adjacent time points exhibit higher Spearman rank correlations than those of randomly paired time points, and the similarity between adjacent Θ further increases with larger values of β, whereas randomly paired Θ show no significant changes. Meanwhile, Fig. 5D (lower panel) demonstrates that increasing either λ or *thre* consistently results in sparser precision matrices, with the sparsity being substantially more sensitive to *thre* than to λ. For the selection of these hyperparameters, one approach is to choose them empirically according to the requirements of a specific problem, while another is to determine them by maximizing the likelihood function, as illustrated in Fig.2G.

The complete regulatory network inferred by GGANO is depicted in Fig. 5E (left panel). Focusing on a core subnetwork comprising four pivotal genes—Nanog, Gata6, Pou5f1, and Cdx2—which are central to cell fate decisions during early mouse embryogenesis (13, 14). By analyzing the evolution of this subnetwork over time (Fig. 5E and Fig. S5), we uncover distinct regulatory patterns associated with the two major fate decisions. During the first cell fate decision, characteristic interactions between Pou5f1 and Cdx2 are observed. Previous studies have demonstrated that the mutual antagonism between Pou5f1 and Cdx2 plays a key role in the establishment and maintenance of the TE lineage (15). In addition, it has been reported that a double negative-feedback loop between Cdx2 and the Pou5f1–Nanog module ensures that Cdx2 expression is restricted to TE cells, while Pou5f1 and Nanog gradually become confined to cells of the ICM (16, 17). These observations are consistent with our results. During the second cell fate decision, we detect reciprocal inhibition between Nanog and Gata6. Prior studies indicate that Nanog and Gata6 are co-expressed during the 8-cell stage and 16-cell stage, subsequently undergo differential up- or down-regulation around the 32-cell stage, and ultimately exhibit mutually repressing expression patterns in the 64-cell stage (17).

9. Biological characterization of the five key EMT-associated genes (TMSB10, MT2A, LGALS1, TGFBI, and OCIAD2)

From the calculation of $Score_{E \to M}$ on the three datasets, we find that in the knockout experiments of both the M and E states, the $Score_{E \to M}$ of genes TMSB10, MT2A, LGALS1, TGFBI, and OCIAD2 were all positive (Fig. 6C). Firstly, regarding the gene TMSB10, it belongs to the β-thymosin family, and the main members of this family also comprise TMSB4 and TMSB15. They can inhibit actin polymerization through binding to β-actin, thereby playing a crucial role in regulating the cytoskeletal microfilament system (18). Moreover, they also possess multiple physiological functions, including tissue development and regeneration, anti-inflammatory actions, and the induction of insulin secretion (18). Recently, there is a growing body of evidence suggesting that TMSB10 is implicated in tumorigenesis and tumor progression. For example, it was found that the expression of TMSB10 was significantly higher in renal cancer cells and tissues than in normal renal cells and tissues, and it regulates the metastasis of malignant cells by inducing EMT (19), and can act as a clinical biomarker for clear cell renal cell carcinoma (20). Further studies have demonstrated that TMSB10 is transcriptionally regulated by JUN, thereby facilitating the proliferation of clear cell renal cell carcinoma cells (21). Meanwhile, TMSB10 can promote the proliferation, invasion, and migration of breast cancer cells via the AKT/FOXO signaling pathway (22). Furthermore, some researchers discovered that TMSB10 is regulated by super enhancers in the EMT of lung adenocarcinoma cells, and proposed that inhibiting the super enhancers regulating TMSB10 might be a novel approach for the treatment of lung adenocarcinoma (23).

The MT2A gene is a member of the metallothionein (MT) family. MT is a cysteine-rich protein that plays a crucial role in metal homeostasis, heavy metal toxicity, DNA damage, and protection against oxidative stress. Abnormal expressions of MT genes have been witnessed in multiple tumors, and this anomaly is closely associated with tumor formation, metastasis potential, the emergence of drug resistance, and poor prognosis (24). Specifically, studies have indicated that MT2A participates in the carcinogenic process of clear cell renal cell carcinoma and regulates the proliferation and migration of malignant cells (25). Further research has disclosed that RING1 can promote the

ubiquitination and degradation of HSF1, which inhibits the transcriptional activation effect of HSF1 on MT2A, thereby resulting in cell cycle arrest and apoptosis in breast cancer cells (26). It is notable that the function of the gene MT2A is related to various factors such as different tumor types, tumor differentiation status, environmental stimuli, and gene mutations. For instance, some studies have demonstrated that MT2A is downregulated in colorectal cancer (26), but some researchers have discovered that MT2A is upregulated in human colorectal cancer HT29 cells (27). Additionally, MT2A is upregulated in osteosarcoma, breast cancer, and prostate cancer (28–30) and has a carcinogenic effect, while it is downregulated in gastric cancer and liver cancer (31, 32) and has a tumor suppressor effect. Then, for LGALS1, it is the first member of the lectin family possessing a carbohydrate recognition structure. When LGALS1 is secreted, it interacts with extracellular matrix glycoproteins like laminin or fibronectin and plays a role in the processes that facilitate tumor cell metastasis, such as cell division, migration, adhesion, invasion, and immune response (33). Previous studies have indicated that the overexpression of LGALS1 can promote the expression of TGF-β1, thereby inducing the EMT in gastric cancer cells (34). Additionally, it has been verified that the overexpression of NCAPG can also promote the EMT of tumor cells (35), and the interaction between NCAPG and LGALS1 can further aggravate the proliferation, invasion, and migration of non-small cell lung cancer cells (36). In the study of melanoma, it was discovered that LGALS1 is the target bound by miR-22-3p, which can inhibit the EMT of melanoma cells and thereby hinder the development of melanoma (37). Not only so, LGALS1 has also been found to induce the EMT in various cancers such as ovarian cancer, liver cancer, and oral cancer (33, 38–41).

Regarding TGFBI and OCIAD2, TGFBI is a type of extracellular matrix protein, also known as BIGH3, which regulates numerous biological functions during embryonic development and the pathogenesis of human diseases, including cell adhesion and bone formation (42). TGFBI has dual effects of tumor suppression and tumor promotion. In the early stage of tumorigenesis, TGFBI can inhibit cell proliferation and induce cell apoptosis, while during the process of cancer progression, TGFBI mainly acts as a tumor promoter (42). Recently, studies have indicated that TGFBI is involved in the EMT of renal cancer and influences the development of renal cancer through the PI3K/AKT/mTOR/HIF-1α signaling pathway (43). Simultaneously, highly expressed TGFBI can

facilitate the EMT, proliferation, and invasive progression of bladder cancer cells (44), and it may also promote tumor growth and drug resistance by influencing the tumor microenvironment of pancreatic cancer (45). Furthermore, TGFBI has also been discovered to play a significant role in tumors such as prostate, ovary, and colon (46–48). OCIAD, as an immune response-related protein, its function remains not fully elucidated, but it is regarded as potentially playing a crucial role in regulating endocytosis and maintaining the homeostasis of hematopoietic stem cells (49). Recently, several studies have reported the possible correlation between OCIAD2 and various cancers. For example, in lung adenocarcinoma, the expression level of OCIAD2 in invasive tumors is significantly higher than that in in situ tumors (50). In ovarian mucinous tumors, the high expression of OCIAD2 is more prevalent in carcinomas and borderline tumors (51). Some researchers have discovered that OCIAD2 is involved in the reconfiguration of the glycolytic metabolic phenotype and can promote the progression of pancreatic adenocarcinoma by activating the AKT signaling pathway (49). In mice, knockdown of miR-145-5p and overexpression of OCIAD2 can facilitate the growth of lung adenocarcinoma tumors (52). Additionally, studies have verified that the TGF signal induces the expression of OCIAD2 via Smad2/3 and Smad4, thereby exerting a role in EMT (53).

In conclusion, the five key genes we predicted that can facilitate the transition from E state to M state have obtained support from the previous literatures. These genes might be significant biomarkers and could also be candidate targets for suppressing EMT, thereby offering potential novel directions for the development of anti-cancer drugs.

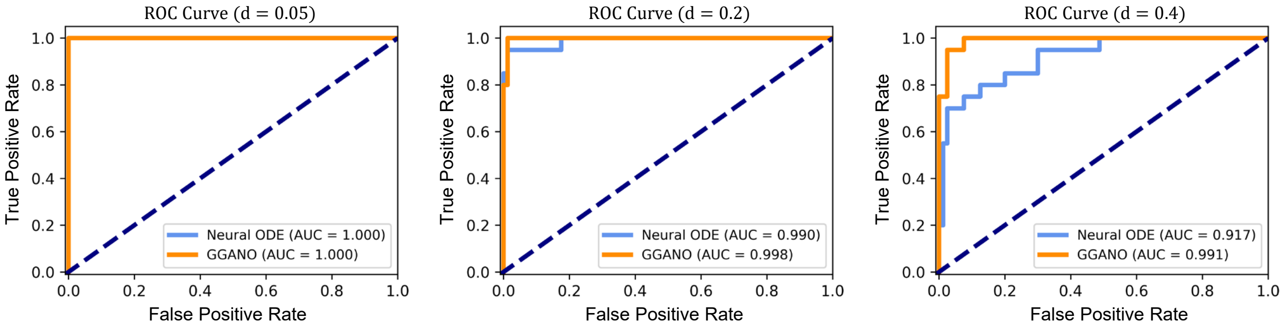

Fig. S1. For the ten-dimensional synthetic model, ROC curves are plotted based on the estimated SM matrices to compare the performance differences between the pure Neural ODE and GGANO in network inference tasks under different noise levels.

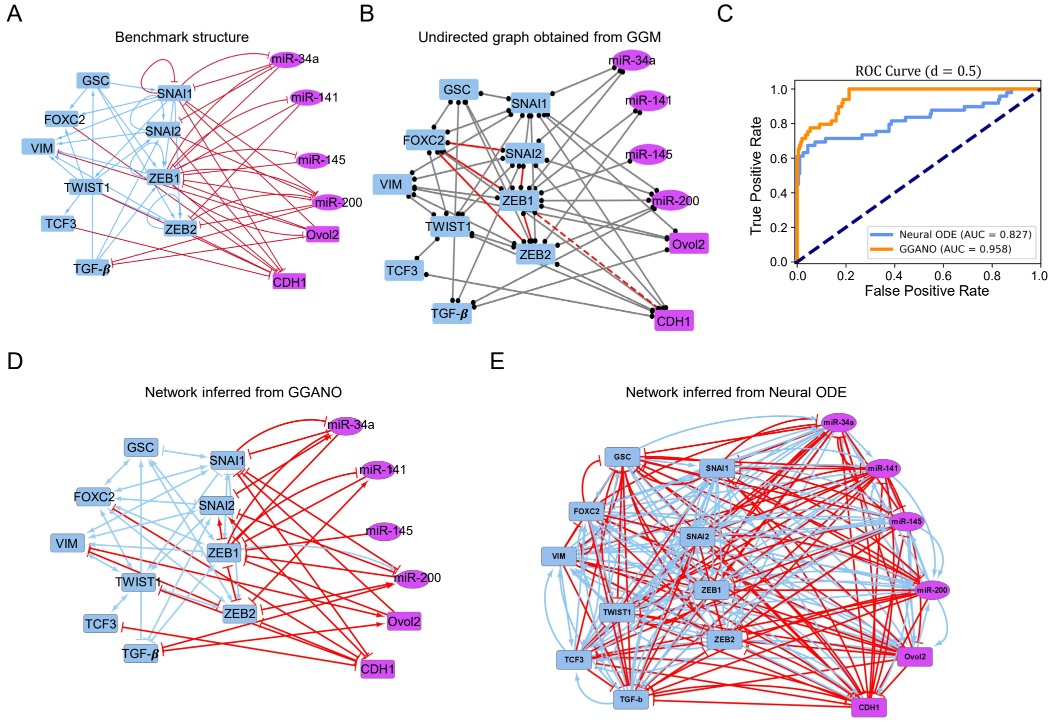

Fig. S2. A The benchmark structure of the network, including 12 transcriptional factors (rectangle nodes), four microRNAs (ellipse nodes), and 53 regulatory links(10). Blue arrows represent activation, and red perpendicular bars represent inhibition. The pink nodes are epithelial markers, and the blue nodes are mesenchymal markers. **B** The undirected graph structure inferred by GGM when the noise level d=0.5. The gray solid lines represent the structure consistent with the standard network in A, while the red solid lines indicate additional predictions, and the missing regulatory interactions are also marked by the red dotted line. **C** ROC curves generated by the two methods. **D** The network inferred by GGANO under d=0.5. **E** The network inferred by pure Neural ODE under d=0.5.

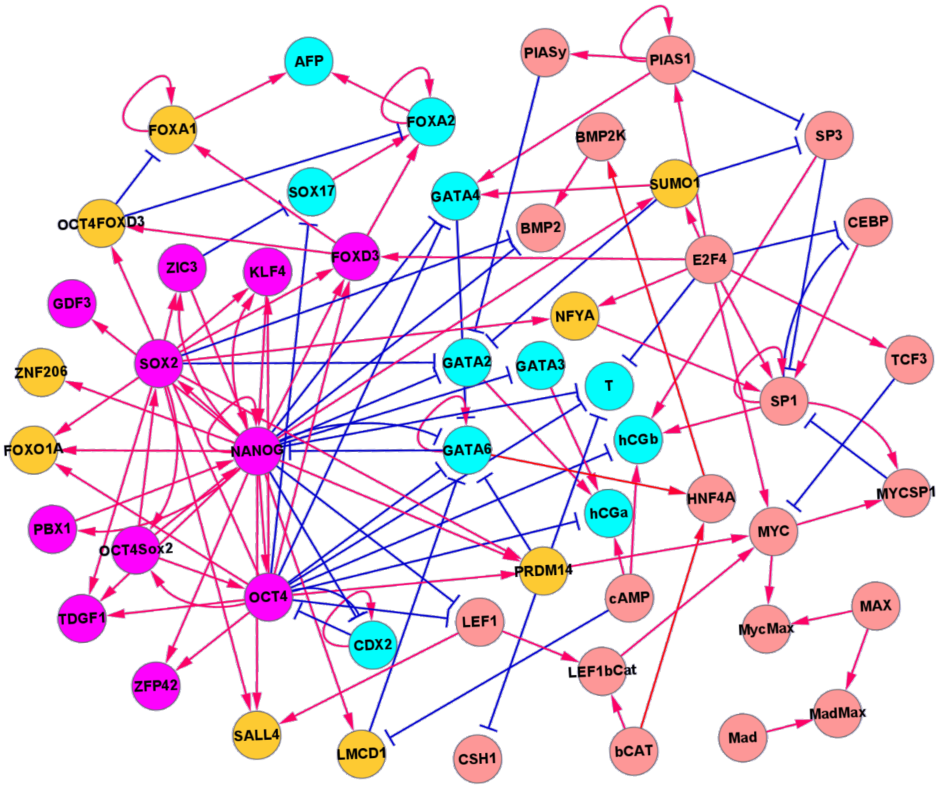

Fig. S3. The diagram for human embryonic stem cell (HESC) network (2). This network includes 52 genes and their interactions (arrows represent activation and perpendicular bars represent repression). The purple nodes represent 11 marker genes for the pluripotent stem cell state, cyan nodes represent 11 marker genes for the differentiation state, the orange nodes represent genes that are activated by the stem cell marker genes, and the light red color nodes denote other genes.

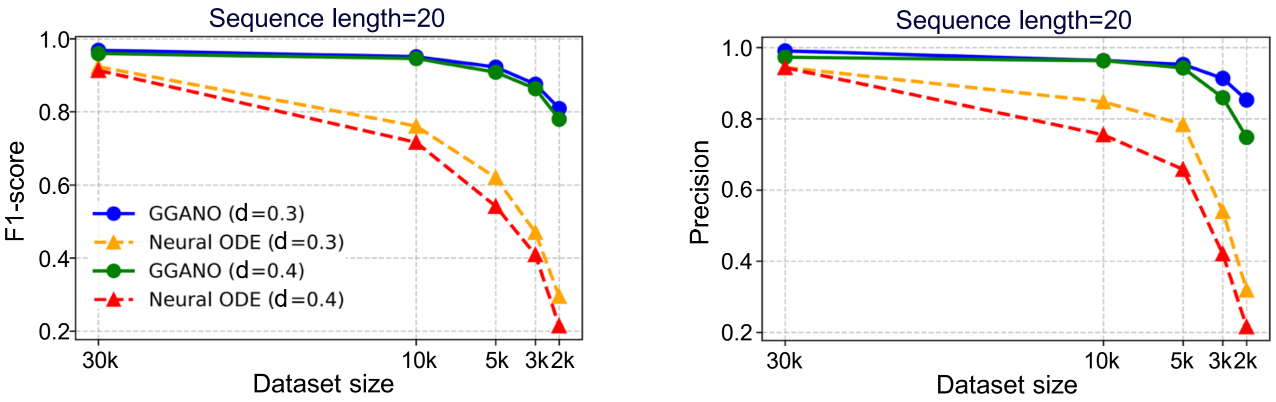

Fig. S4. Comparison of F1-score and Precision for the pure Neural ODE and GGANO as the training set size decreases, with fixed sequence lengths of 20.

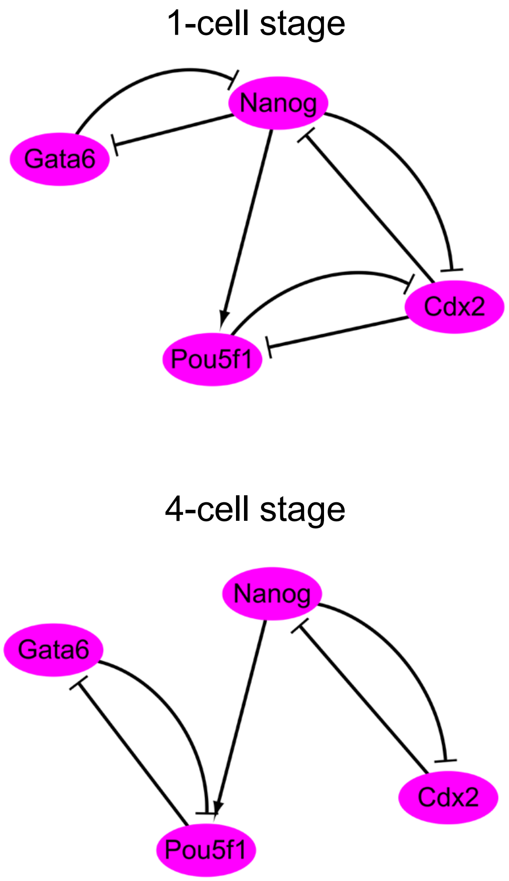

Fig. S5. The temporal changes of the key subnetwork from the 1-cell to the 8-cell stage.

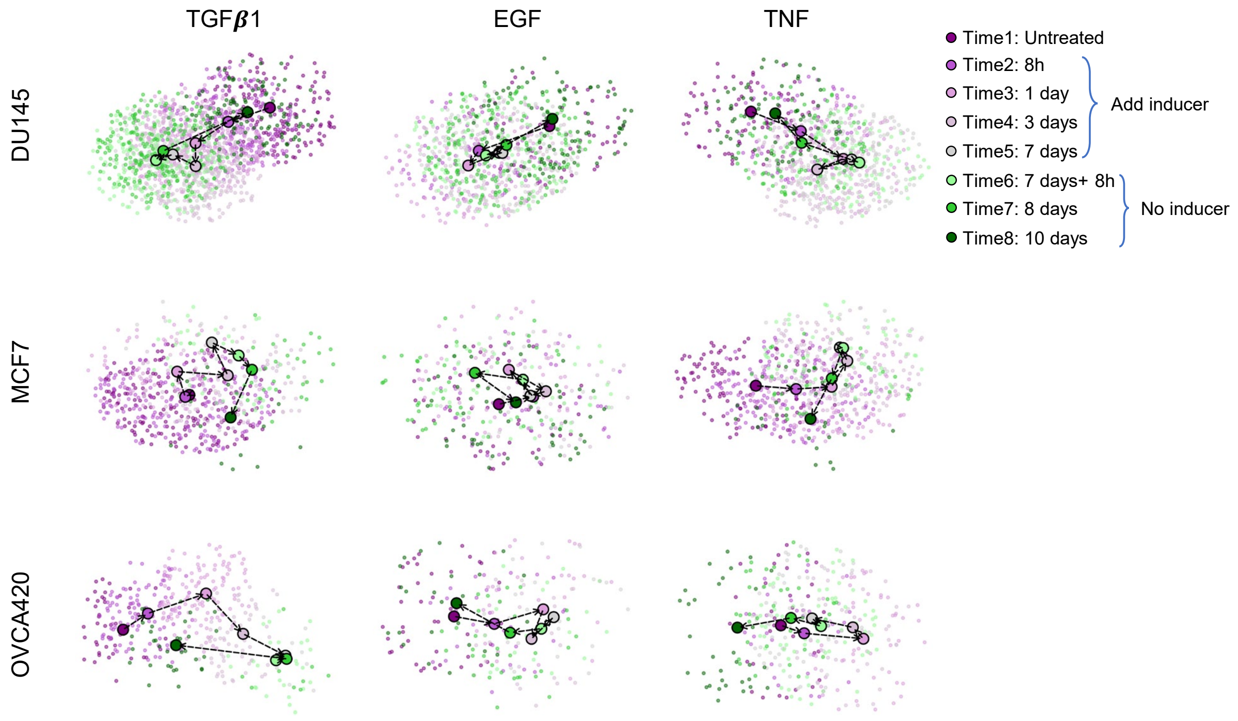

Fig. S6. PCA embeddings of the EMT time-course experiments performed in the other three cell lines. Each small point represents an individual sample, while larger points indicate the average of all data points at that time point.

The network inferred from data with the inducer EGF

The network inferred from data with the inducer TNF

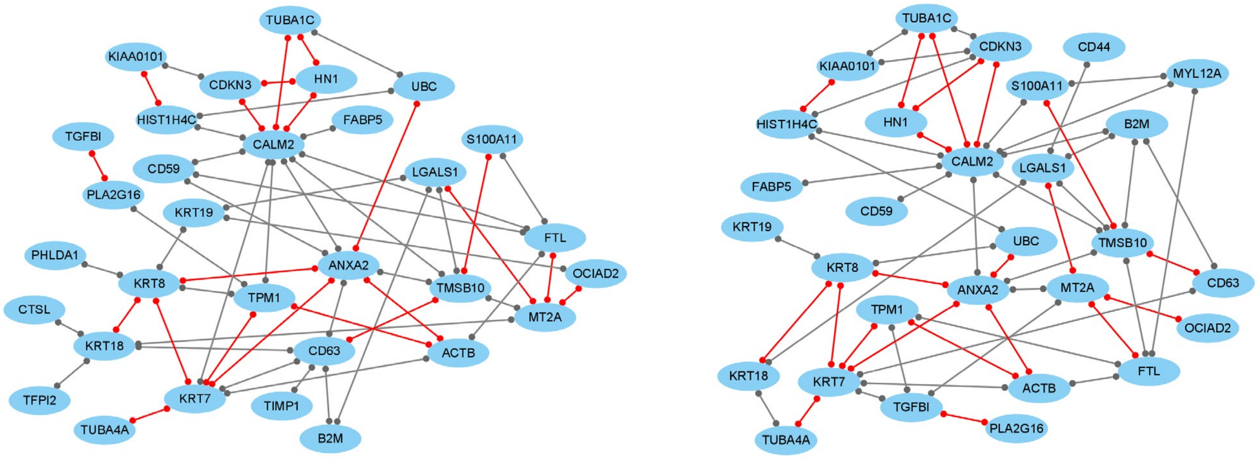

Fig. S7. The undirected graph structures inferred by the GGM from A549 cell line data with EGF and TNF inducers. Red edges indicate regulatory interactions shared across all three networks.

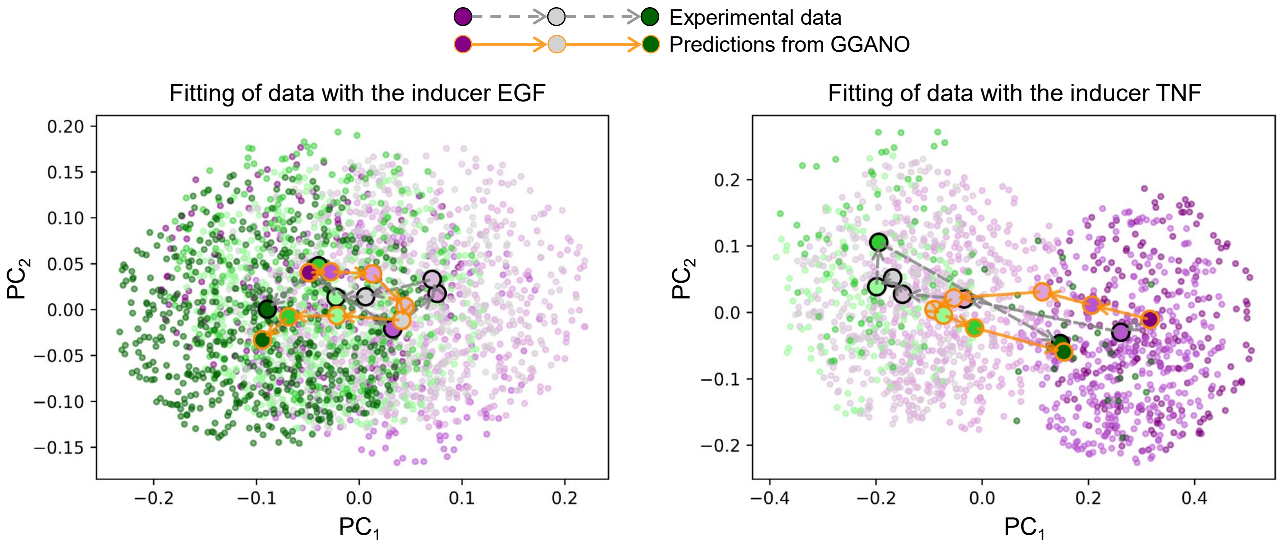

Fig. S8. The fitting performance of GGANO on experimental data. The orange line represents the predictions from GGANO, while the gray line represents the experimental data.

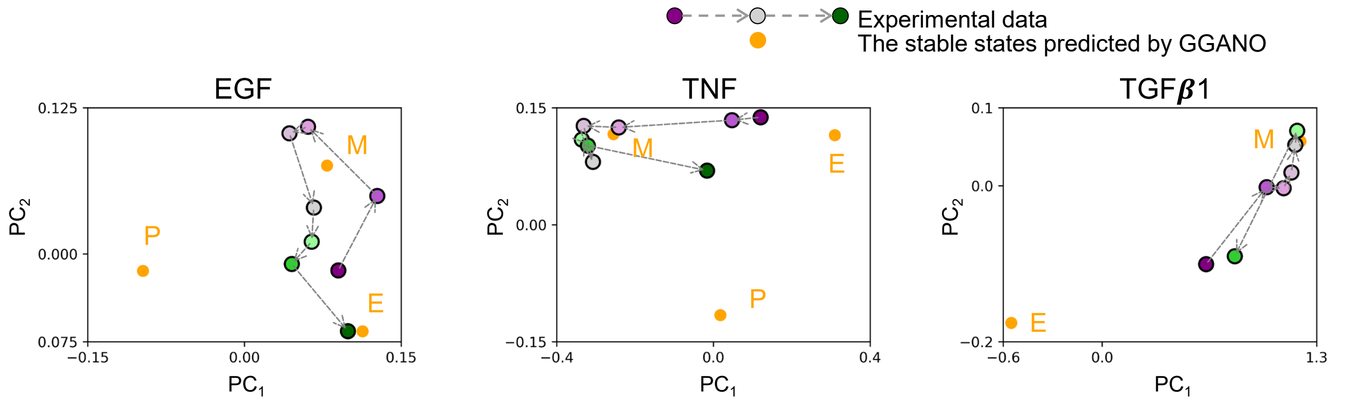

Fig. S9. Comparison of the GGANO-predicted steady states and experimental data. The M state generated by GGANO is highly consistent with the data at the fifth time point in the three datasets. However, for the TGFβ1 and TNF data, a certain deviation exists between the E state generated by GGANO and the corresponding experimental data at the first and eighth time points.

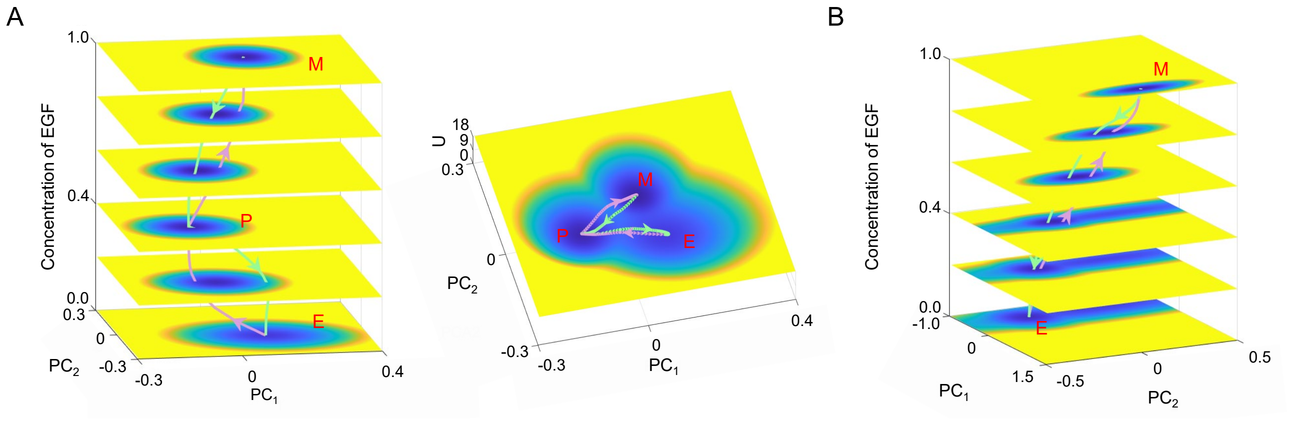

Fig. S10. A The potential energy landscape of the system at different concentrations of the inducer TNF. When the concentration is 0.0, the system is in the E state, and as the concentration increases, the system gradually transitions from the E state to the M state. When the concentration reaches 1.0, the system is in the M state, and as the concentration decreases, the system returns from the M state to the E state. The purple line represents the transition path from the E state to the M state, while the green line represents the reverse transition from the M state to the E state. E: Epithelial state, M: Mesenchymal state, P: Partial EMT state. **B** The potential energy landscape of the system at different concentrations of the inducer TGFβ1.

References

1. Cook D P, Vanderhyden B C. Context specificity of the EMT transcriptional response. *Nature communications*. 2020; 11(1): 2142.
2. Li C, Wang J. Quantifying cell fate decisions for differentiation and reprogramming of a human stem cell network: landscape and biological paths. *PLoS computational biology*. 2013; 9(8): e1003165.
3. Hill A V. The possible effects of the aggregation of the molecules of hemoglobin on its dissociation curves. *The Journal of Physiology*. 1910; 40: 4-7.
4. Yu L, Lu M, Jia D, Ma J, Ben-Jacob E, Levine H, et al. Modeling the genetic regulation of cancer metabolism: interplay between glycolysis and oxidative phosphorylation. *Cancer research*. 2017; 77(7): 1564-1574.
5. Sisan D R, Halter M, Hubbard J B, Plant A L. Predicting rates of cell state change caused by stochastic fluctuations using a data-driven landscape model. *Proceedings of the National Academy of Sciences*. 2012; 109(47): 19262-19267.
6. Hu G. Stochastic forces and nonlinear systems. *Shanghai Scientific and Technological Education Publishing House, Shanghai*. 1994; p. 68-74.
7. Van Kampen N G. Stochastic Processes in Physics and Chemistry. *3rd ed. Amsterdam: North Holland*. 2007.
8. Kang X, Li C. A dimension reduction approach for energy landscape: identifying intermediate states in metabolism-EMT network. *Advanced Science*. 2021; 8(10): 2003133.
9. Li C, Wang J. Landscape and flux reveal a new global view and physical quantification of mammalian cell cycle. *Proceedings of the National Academy of Sciences*. 2014; 111(39): 14130-14135.
10. Lang J, Nie Q, Li C. Landscape and kinetic path quantify critical transitions in epithelial-mesenchymal transition. *Biophysical Journal*. 2021; 120(20): 4484-4500.
11. Li C, Hong T, Nie Q. Quantifying the landscape and kinetic paths for epithelial–mesenchymal transition from a core circuit. *Physical Chemistry Chemical Physics* 2016; 18(27): 17949-17956.

12. Guo G, Huss M, Tong G Q, Wang C, Sun L L, Clarke N D, et al. Resolution of cell fate decisions revealed by single-cell gene expression analysis from zygote to blastocyst. *Developmental cell*. 2010; 18(4): 675-685.
13. Li C, Wang J. Quantifying Waddington landscapes and paths of non-adiabatic cell fate decisions for differentiation, reprogramming and transdifferentiation. *Journal of The Royal Society Interface*. 2013; 10(89): 20130787.
14. Allègre N, Chauveau S, Dennis C, Renaud Y, Meistermann D, Estrella L V, et al. NANOG initiates epiblast fate through the coordination of pluripotency genes expression. *Nature Communications*. 2022; 13(1): 3550.
15. Niwa H, Toyooka Y, Shimosato D, Strumpf D, Takahashi K, Yagi R, Rossant J. Interaction between Oct3/4 and Cdx2 determines trophectoderm differentiation. *Cell*. 2005; 123(5): 917-929.
16. Strumpf D, Mao C A, Yamanaka Y, Ralston A, Chawengsaksophak K, Beck F, et al. Cdx2 is required for correct cell fate specification and differentiation of trophectoderm in the mouse blastocyst. *Development*. 2005; 132 (9): 2093–2102.
17. Takaoka K, Hamada H. Cell fate decisions and axis determination in the early mouse embryo. *Development*. 2012; 139(1): 3-14.
18. Sribenja S, Wongkham S, Wongkham C, Yao Q, Chen C. Roles and mechanisms of β-thymosins in cell migration and cancer metastasis: an update. *Cancer investigation*. 2013; 31(2): 103-110.
19. Xiao R, Shen S, Yu Y, Pan Q, Kuang R, Huang H. TMSB10 promotes migration and invasion of cancer cells and is a novel prognostic marker for renal cell carcinoma. *International journal of clinical and experimental pathology*. 2019; 12(1): 305.
20. Pan Q, Cheng G, Liu Y, Xu T, Zhang H, Li B. TMSB10 acts as a biomarker and promotes progression of clear cell renal cell carcinoma. *International journal of oncology*. 2020; 56(5): 1101-1114.
21. Wang C, He Y, You Z, Chen X. TMSB10 promotes progression of clear cell renal cell carcinoma via JUN transcription regulation. *Annals of Clinical & Laboratory Science*. 2022; 52(2): 230-239.
22. Zhang X, Ren D, Guo L, Wang L, Wu S, Lin C, et al. Thymosin beta 10 is a key regulator of tumorigenesis and metastasis and a novel serum marker in breast cancer. *Breast Cancer*

Research. 2017; 19: 1-15.

23. Liu Y. Identification and comprehensive analysis of super-enhancer related genes involved in epithelial-to-mesenchymal transition in lung adenocarcinoma. *PloS One*. 2023; 18(9): e0291088.
24. Liu X, Quan J, Shen Z, Zhang Z, Chen Z, Li L, et al. Metallothionein 2A (MT2A) controls cell proliferation and liver metastasis by controlling the MST1/LATS2/YAP1 signaling pathway in colorectal cancer. *Cancer Cell International*. 2022; 22(1): 205.
25. Wang J, Zuo Z, Yu Z, Chen Z, Meng X, Ma Z, et al. Single-cell transcriptome analysis revealing the intratumoral heterogeneity of ccRCC and validation of MT2A in pathogenesis. *Functional & Integrative Genomics*. 2023; 23(4): 300.
26. Liu D, Guo Y, Du Q, Zhu Y, Guo Y. RING induces cell cycle arrest and apoptosis in human breast cancer cells by regulating the HSF1/MT2A axis. *Experimental Cell Research*. 2023; 433(1): 113795.
27. Fűri I, Kalmár A, Wichmann B, Spisák S, Schöller A, Barták B, et al. Cell free DNA of tumor origin induces a 'metastatic'expression profile in HT-29 cancer cell line. *PLoS One*. 2015; 10(7): e0131699.
28. Zaman M S, Barman S K, Corley S M, Wilkins M R, Malladi C S, Wu M J. Transcriptomic insights into the zinc homeostasis of MCF-7 breast cancer cells via next-generation RNA sequencing. *Metallomics*. 2021; 13(6): mfab026.
29. Habel N, Hamidouche Z, Girault I, Patino-Garcia A, Lecanda F, Marie P J, et al. Zinc chelation: a metallothionein 2A's mechanism of action involved in osteosarcoma cell death and chemotherapy resistance. *Cell death & disease*. 2013; 4(10): e874-e874.
30. Ma D, Zhou Z, Yang B, He Q, Zhang Q, Zhang X H. Association of molecular biomarkers expression with biochemical recurrence in prostate cancer through tissue microarray immunostaining. *Oncology Letters*. 2015; 10(4): 2185-2191.
31. Ullio C, Brunk U T, Urani C, Melchioretto P, Bonelli G, Baccino F M, et al. Autophagy of metallothioneins prevents TNF-induced oxidative stress and toxicity in hepatoma cells. *Autophagy*. 2015; 11(12): 2184-2198.
32. Tong M, Lu W, Liu H, Wu J, Jiang M, Wang X, et al. Evaluation of MT family isoforms as

potential biomarker for predicting progression and prognosis in gastric cancer. *BioMed Research International*. 2019; 2019(1): 2957821.

33. Li X, Wang H, Jia A, Cao Y, Yang L, Jia Z. LGALS1 regulates cell adhesion to promote the progression of ovarian cancer. *Oncology Letters*. 2023; 26(2): 1-11.
34. Wu J, Zhao X, Jiang X, Tao W, Chen Z, Huang C, et al. Galectin-1 From Cancer-Associated Fibroblasts Promotes the Invasion and Metastasis of Gastric Cancer Through TGF-β1-Induced Epithelial-Mesenchymal Transition. 2021.
35. Zhang X, Zhu M, Wang H, Song Z, Zhan D, Cao W, et al. Overexpression of NCAPG inhibits cardia adenocarcinoma apoptosis and promotes epithelial-mesenchymal transition through the Wnt/β-catenin signaling pathway. *Gene*. 2021; 766: 145163.
36. Sun H, Zhang H, Yan Y, Li Y, Che G, Zhou C, et al. NCAPG promotes the oncogenesis and progression of non-small cell lung cancer cells through upregulating LGALS1 expression. *Molecular cancer*. 2022; 21(1): 55.
37. Chen Y, Fang Y, Li L, Luo H, Cao T, Tu B. Exosomal miR-22-3p from mesenchymal stem cells inhibits the epithelial-mesenchymal transition (EMT) of melanoma cells by regulating LGALS1. *Frontiers in Bioscience-Landmark*. 2022; 27(9): 275.
38. Liu Y, Wu L, Ao H, Zhao M, Leng X, Liu M, et al. Prognostic implications of autophagy-associated gene signatures in non-small cell lung cancer. *Aging (albany NY)*. 2019; 11(23): 11440.
39. Zhang P F, Li K S, Shen Y H, Gao P T, Dong Z R, Cai J B, et al. Galectin-1 induces hepatocellular carcinoma EMT and sorafenib resistance by activating FAK/PI3K/AKT signaling. *Cell death & disease*. 2016; 7(4): e2201.
40. Zhu J, Zheng Y, Zhang H, Liu Y, Sun H, Zhang P. Galectin-1 induces metastasis and epithelial-mesenchymal transition (EMT) in human ovarian cancer cells via activation of the MAPK JNK/p38 signalling pathway. *American journal of translational research*. 2019; 11(6): 3862.
41. Li J M, Tseng C W, Lin C C, Law C H, Chien Y A, Kuo W H, et al. Upregulation of LGALS1 is associated with oral cancer metastasis. *Therapeutic Advances in Medical Oncology*. 2018; 10: 1758835918794622.

42. Corona A, Blobe G C. The role of the extracellular matrix protein TGFBI in cancer. *Cellular signalling*. 2021; 84: 110028.

43. Zhan S, Bai X, Zhao Y, Tuoheti K, Yisha Z, Zuo Y, et al. TGFBI promotes proliferation and epithelial–mesenchymal transition in renal cell carcinoma through PI3K/AKT/mTOR/HIF-1α pathway. *Cancer Cell International*. 2024; 24(1): 265.

44. Zou J, Huang R, Li H, Wang B, Chen Y, Chen S, et al. Secreted TGF-beta-induced protein promotes aggressive progression in bladder cancer cells. *Cancer management and research*. 2019; 6995-7006.

45. Zhou J, Lyu N, Wang Q, Yang M, Kimchi E T, Cheng K, et al. A novel role of TGFBI in macrophage polarization and macrophage-induced pancreatic cancer growth and therapeutic resistance. *Cancer letters*. 2023; 578: 216457.

46. Chen W Y, Tsai Y C, Yeh H L, Suau F, Jiang K C, Shao A N, et al. Loss of SPDEF and gain of TGFBI activity after androgen deprivation therapy promote EMT and bone metastasis of prostate cancer. *Science signaling*. 2017; 10(492): eaam6826.

47. Lecker L S M, Berlato C, Maniati E, Delaine-Smith R, Pearce O M, Heath O, et al. TGFBI production by macrophages contributes to an immunosuppressive microenvironment in ovarian cancer. *Cancer research*. 2021; 81(22): 5706-5719.

48. Jiang R, Chen X, Ge S, Wang Q, Liu Y, Chen H, et al. MiR-21-5p induces pyroptosis in colorectal cancer via TGFBI. *Frontiers in oncology*. 2021; 10: 610545.

49. Yin Y F, Jia Q Y, Yao H F, Zhu Y H, Zheng J H, Duan Z H, et al. OCIAD2 promotes pancreatic cancer progression through the AKT signaling pathway. *Gene*. 2024; 927: 148735.

50. Ishiyama T, Kano J, Anami Y, Onuki T, Iijima T, Morisita Y, et al. OCIA domain containing 2 is highly expressed in adenocarcinoma mixed subtype with bronchioloalveolar carcinoma component and is associated with better prognosis. *Cancer science*. 2007; 98(1): 50-57.

51. Nagata C, Kobayashi H, Sakata A, Satomi K, Minami Y, Morishita Y, et al. Increased expression of OCIA domain containing 2 during stepwise progression of ovarian mucinous tumor. *Pathology International*. 2012; 62(7): 471-476.

52. He Y, Li G, Fu R, Li Y, Wang Y. Hsa_circ_0001492 regulates the hsa-miR-145-5p/ovarian carci-

noma immunoreactive antigen domain 2 axis to promote the progression of lung adenocarcinoma. *Biomolecules and Biomedicine*. 2024.

53. Zhang R, Zhao C, Xiong Z, Zhou X. Pathway bridge based multiobjective optimization approach for lurking pathway prediction. *BioMed Research International*. 2014; 2014(1): 351095.
